# SAR202 Genomes from the Dark Ocean Predict Pathways for the Oxidation of Recalcitrant Dissolved Organic Matter

**DOI:** 10.1128/mBio.00413-17

**Published:** 2017-04-18

**Authors:** Zachary Landry, Brandon K. Swan, Gerhard J. Herndl, Ramunas Stepanauskas, Stephen J. Giovannoni

**Affiliations:** a Department of Microbiology, Oregon State University, Corvallis, Oregon, USA; b Bigelow Laboratory for Ocean Sciences, Single-Cell Genomics Center, East Boothbay, Maine, USA; c National Biodefense Analysis and Countermeasures Center, Frederick, Maryland, USA; d Department of Marine Biology, University of Vienna, Vienna, Austria; e Department of Marine Microbiology and Biogeochemistry, NIOZ, Royal Netherlands Institute for Sea Research, Utrecht University, Texel, The Netherlands; University of Oklahoma

**Keywords:** bathypelagic, *Chloroflexi*, dissolved organic matter, mesopelagic, monooxygenase, SAR202, single-cell genomics

## Abstract

Deep-ocean regions beyond the reach of sunlight contain an estimated 615 Pg of dissolved organic matter (DOM), much of which persists for thousands of years. It is thought that bacteria oxidize DOM until it is too dilute or refractory to support microbial activity. We analyzed five single-amplified genomes (SAGs) from the abundant SAR202 clade of dark-ocean bacterioplankton and found they encode multiple families of paralogous enzymes involved in carbon catabolism, including several families of oxidative enzymes that we hypothesize participate in the degradation of cyclic alkanes. The five partial genomes encoded 152 flavin mononucleotide/F420-dependent monooxygenases (FMNOs), many of which are predicted to be type II Baeyer-Villiger monooxygenases (BVMOs) that catalyze oxygen insertion into semilabile alicyclic alkanes. The large number of oxidative enzymes, as well as other families of enzymes that appear to play complementary roles in catabolic pathways, suggests that SAR202 might catalyze final steps in the biological oxidation of relatively recalcitrant organic compounds to refractory compounds that persist.

## IMPORTANCE

Carbon in the ocean is massively sequestered in a complex mixture of biologically refractory molecules that accumulate as the chemical end member of biological oxidation and diagenetic change. However, few details are known about the biochemical machinery of carbon sequestration in the deep ocean. Reconstruction of the metabolism of a deep-ocean microbial clade, SAR202, led to postulation of new biochemical pathways that may be the penultimate stages of DOM oxidation to refractory forms that persist. These pathways are tied to a proliferation of oxidative enzymes. This research illuminates dark-ocean biochemistry that is broadly consequential for reconstructing the global carbon cycle.

## INTRODUCTION

The mass of the accumulated refractory dissolved organic matter (DOM) pool in the ocean is nearly equivalent to the carbon in atmospheric CO_2_, making it an important component of the global carbon budget (1). Deep-ocean DOM is composed of a heterogeneous mixture of carbon compounds that are thought mainly to originate from photosynthesis and carbon cycling activity in the upper ocean (the euphotic zone and the upper mesopelagic) (1–4). Persistent deep-ocean DOM, which can have a half-life of thousands of years, is thought to be either intrinsically refractory to biological oxidation (5) or to yield too little energy to benefit cells, presumably because the cellular cost of synthesizing the enzymes needed to catabolize it exceeds the value of the resource (6–8). Some data suggest that a fraction of the DOM, resistant to biological oxidation at low concentrations, can become labile at higher concentrations (8). In any case, the biological reactivity of DOM is a continuum, with labile DOM (operationally defined as having turnover times from hours to days) at one end of the spectrum, to recalcitrant compounds (defined as having turnover times from decades to centuries), and finally the refractory end members that persist in the environment for millennia (3). Both biotic and abiotic factors complicate DOM diagenesis: molecules can be altered by heterotrophic microbes that use the most reactive moieties to provide carbon and energy, and molecular heterogeneity can be increased by abiotic transformations, such as racemization. As labile DOM is converted to refractory DOM by heterotrophic marine bacteria, they produce inorganic carbon and new biomass, some fraction of which is also recalcitrant or refractory. The microbial carbon pump (MCP) describes the combined effect of these processes, which is a net sequestration of organic carbon resulting from the conversion of the labile organic carbon pool to a biologically recalcitrant mixture (2, 4, 8, 9). However, the MCP is largely conceptual and is built from concepts that are illustrated by a few examples, with most microbiological and biochemical details unknown.

SAR202 is a clade of uncultured bacteria that were first discovered in 1993 in the mesopelagic ocean (200 to 1,000 m) (10). Later studies confirmed these bacteria are rare in sunlit surface waters but comprise about 10% of all plankton cells in the dark ocean, where they increase from about 5% of mesopelagic communities to up to 30% in the bathypelagic (1,000 to 5,000 m) (11–14). They have since been found to be ubiquitous in the dark ocean, as well as common in subseafloor environments and deep lakes (15–17). The metabolism and geochemical role of the SAR202 clade are unknown, but their high abundance and evolutionary position in the phylum *Chloroflexi* have long fueled speculation they might play a role in the degradation of recalcitrant organic matter (10, 14).

The *Chloroflexi* have an ancient origin among the early diverging lineages of the domain *Bacteria* (18–21). Current estimates place the branching of the *Chloroflexi* at ~2.8 billion years ago (Ga), during the Early Proterozoic (2.5 to 0.5 Ga), a period that coincides with the rapid appearance of the first oxygen-consuming families of enzymes (22). SAR202 encompasses a diverse monophyletic group of lineages that are nearly all planktonic or associated with sediment in deep-ocean and deep-lake environments. Although there is great uncertainty about early events in bacterial evolution, a simple hypothesis that is consistent with the modern ecology of the SAR202 clade and insights from phylogenetic inference is that SAR202 diversified into dark-ocean niches sometime in the Precambrian eon (4.5 to 0.5 Ga).

The phylum *Chloroflexi* encompasses a tremendously wide range of metabolic strategies, from anoxygenic photosynthesis to anaerobic reductive dehalogenation in *Dehalococcoides*, which has an extremely small genome size of 1.4 Mbp (23), to aerobic heterotrophy in the filamentous, sporeformer *Ktedonobacter racemifer* (24, 25), which has one of the largest sequenced bacterial genomes (13 Mbp). Metabolic diversity within *Chloroflexi* has precluded predictions about the geochemical activity of the SAR202 clade from phylogeny alone, and thus far these cells have eluded cultivation.

Although deep-ocean DOM is thought mainly to consist of refractory compounds (3) that have an estimated turnover time of ~30,000 years, it has been proposed that about 30% of deep-ocean DOM is semilabile, with a turnover time of <50 years (26). The slow oxidation of this DOM fraction is likely to be a source of energy for specialized deep-ocean microbial communities, along with chemolithotrophic metabolism that is based primarily on inorganic compounds that also originate from DOM. Although deep-ocean DOM is highly heterogeneous, a large fraction of it is thought to consist of cyclic alkanes, the best-documented example of which is known as carboxyl-rich alicyclic matter (CRAM) (27), a mixture of at least several hundred distinct aliphatic ring structures that are rich in oxygenated side groups (27, 28). Nuclear magnetic resonance (NMR) analysis of CRAM indicated that sterol and hopanoid-like ring structures would satisfy the median chemical constraints provided by the NMR data and are likely precursors to some forms of deep-ocean DOM (27). These structures are thought to be extremely stable; hopanoids, for example, are some of the most abundant molecular “fossils” found on earth (29, 30). The natural recalcitrance of cyclic alkanes is likely an additional factor that contributes to the slow oxidation of deep-sea DOM (27, 29).

In this study, we reconstruct SAR202 metabolism from genomes that were acquired from deep-ocean samples by single-cell genome sequencing. We sought to understand biogeochemical functions that could explain the extraordinary success of these cells in the dark ocean. These genome data are made more interesting by the deep-branching position of SAR202 in the tree of life. The early divergence of these cells and their modern distributions together suggest these organisms may have originated in ancient oceans. The unusual complement of genes in these genomes was resolved by reconstructing previously unknown metabolic pathways for DOM catabolism that we hypothesize are active in recalcitrant DOM oxidation. Given the ubiquity and abundance of the SAR202 clade throughout the deep oceans of the world, insight into the metabolism of this group of organisms has far-reaching implications, extending well into the realm of global biogeochemistry.

## RESULTS AND DISCUSSION

### SAG isolation, sequencing, and assembly.

**FIG 1:**
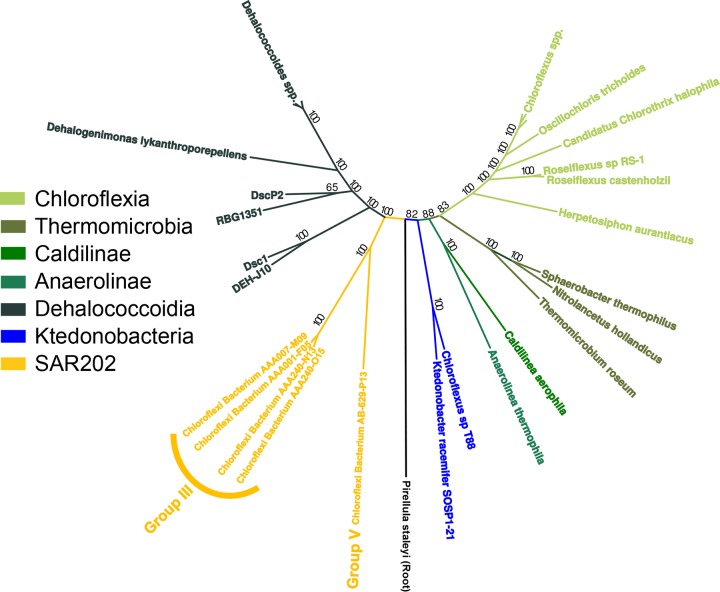
Phylogenomic tree of the *Chloroflexi*. This is a maximum likelihood tree based on a concatenated sequence alignment of 70 amino acid sequences and allowing for 20% missing data. Branches are color coded by class-level taxon assignments. In this tree, the SAR202 clade and *Ktedonobacteria* are the two deepest-branching classes within the phylum. The tree is rooted using *Pirellula staleyi* as an outgroup.

Four SAR202 single-amplified genomes (SAGs) from the group III SAR202 subclade (12) previously shown to be abundant in the mesopelagic zone were initially identified to determine the phylogenetic affiliation of the SAG sequences based on 16S ribosomal RNA sequences (see Fig. S1 in the supplemental material). Two of these SAGs, *Chloroflexi* bacterium SCGC AAA240-N13 and *Chloroflexi* bacterium SCGC AAA240-O15, were isolated from 770 m at the Hawaii Ocean Time-Series (HOTS) sampling site (22°45′N, 158°00′W). The remaining two, *Chloroflexi* bacterium SCGC AAA001-F05 and *Chloroflexi* bacterium SCGC AAA007-M09, were isolated from a depth of 800 m in the South Atlantic Gyre (12°29′S, 4°59′W). A fifth SAG (*Chloroflexi* bacterium SCGC AB-629-P13), belonging to a novel group of SAR202, isolated from the North Atlantic at a depth of 511 m, was chosen for its distant position within the SAR202 clade and will be identified here as the group V SAR202 (Fig. 1; Fig. S1), related to the previously unclassified clone SAR242 described by Morris et al. (12). Assemblies of The SAR202 group III SAGs had total assembly sizes ranging from 1,096,525 bp to 1,423,799 bp. The single group V SAR202 (*Chloroflexi* bacterium SCGC AB-629-P13) had a total assembly size of 807,656 bp. All assemblies from the group III SAR202 SAGs had a consistent GC content of ~55%; the group V SAR202 had a GC content of 41%. Full assembly statistics, potential contamination, and completeness estimates from CheckM and crossover point (Cp) values for all SAGs are included in Table S1 in the supplemental material.

10.1128/mBio.00413-17.2 FIG S1  16S ribosomal RNA-based phylogenetic tree showing the position of the 5 SAR202 SAGs within the phylum *Chloroflexi*. The tree shows 5 distinct groups within the SAR202 clade, the first four of which correspond to the taxonomy put forth by Morris et al. (12). The majority of marine SAR202 members are represented by groups II and III, with the namesake of the clade being placed in group III. Group V represents a previously unreported group of SAR202 whose environmental relevance is yet to be determined. The SAR202 single-amplified genomes (SAGs) are marked in red, with 4 of the genomes belonging to the group III SAR202 and a fifth genome belonging to the group V scale. The tree is rooted on *Streptomyces bikiniensis*. The bar represents 0.2 nucleotide substitution/per site. Download FIG S1, PDF file, 0.1 MB. Copyright © 2017 Landry et al. 2017 Landry et al. This content is distributed under the terms of the Creative Commons Attribution 4.0 International license.

10.1128/mBio.00413-17.8 TABLE S1  Assembly statistics for 5 SAR202 single-amplified genomes (SAGs). The crossover point (Cp) represents the number of hours into the MDA reaction where the product reached half of its maximum double-stranded DNA (dsDNA) fluorescence. Only contigs larger than 2,000 bp were used in our analyses. Download TABLE S1, PDF file, 0.1 MB. Copyright © 2017 Landry et al. 2017 Landry et al. This content is distributed under the terms of the Creative Commons Attribution 4.0 International license.

### Phylogenomic relationships.

In maximum likelihood phylogenomic trees based on 17,003 informative amino acid positions, SAR202 and the *Ktedonobacteria* emerged as the lineages most closely related to the common ancestor of the phylum *Chloroflexi* (Fig. 1). A basal phylogenomic position for *Ktedonobacteria* within the *Chloroflexi* has been observed previously in phylogenomic studies (20, 21). *Ktedonobacter racemifer* was originally isolated from a compost heap using a buffered medium of humic acids and mineral salts (24, 31). Subsequently, this and other *Ktedonobacteria* were shown to be aerobic carbon monoxide oxidizers (32) or chemoheterotrophs capable of utilizing a wide variety of organic carbon compounds (24, 33). The SAR202 group V genome branched deeply within the SAR202 clade, as expected. The closest derived relatives of SAR202 in the tree were *Dehalococcoidia*. Cultured *Dehalococcoidia* are anaerobes that link the oxidation of hydrogen to the reduction of organohalogens (34–38) and also use aromatics and organosulfur and inorganic sulfur compounds as the substrates for energy and growth (39, 40).

### Estimated genome sizes.

Estimated genome sizes are shown in Table 1. Estimation of genome sizes is challenging due to the large evolutionary distances between SAR202 genomes and their most closely related reference genomes. The estimated genome sizes varied widely among the SAGs, ranging from 1.4 to 13.2 Mbp, raising the possibility that these organisms have highly plastic genomes, a trait that has been alluded to in other members of the phylum (25, 41, 42). Genome sizes were estimated from a set of 71 single-copy gene clusters conserved in *Chloroflexi*. A power law regression that assumed the 25 fully sequenced *Chloroflexi* genomes are representative of the phylum indicated that the set of 71 conserved genes is on an asymptote approaching the true core genome set for *Chloroflexi* (see Fig. S2 in the supplemental material). Among the four SAR202 group III SAGs analyzed, estimated genome sizes ranged from ~3 to 13 Mbp. The completeness estimates from this analysis are slightly higher than size estimates generated using CheckM (Table S1). These genome size estimates should be viewed with a degree of caution, as some may overestimate the actual genome size. Outliers in genome size estimation appear to be more prevalent, with both low and high numbers of recovered conserved genes (see Fig. S3 in the supplemental material). Since the genome size estimates for the group III SAR202 genomes appear to converge on 3 to 4 Mbp with increasing levels of completeness, we predict these genomes are most likely in this size range.

10.1128/mBio.00413-17.3 FIG S2  Mean, mode, standard deviation, and power law regression of the distribution of conserved gene set size for all possible combinations of a given number of the *Chloroflexi* genomes included in our analysis. The mode closely follows the values predicted by the power law regression, which approaches an asymptote at 71 conserved genes in the tail region. Download FIG S2, PDF file, 0.1 MB. Copyright © 2017 Landry et al. 2017 Landry et al. This content is distributed under the terms of the Creative Commons Attribution 4.0 International license.

10.1128/mBio.00413-17.4 FIG S3  Box plot distributions of genome recovery as a function of recovered *Chloroflexi*-conserved single-copy marker genes, based on a rolling starting point within the concatenated genome sequence for all *Chloroflexi* included in our analysis. The boxes represent the first and third quartiles of genome fractions recovered for a given number of recovered conserved single-copy genes. Whiskers represent the interquartile range × 1.5 (IQR 1.5) of genome fractions recovered for a given number of recovered conserved single-copy genes. Red bars represent the median, and “+” symbols represent outlier data points outside the IQR 1.5. Notice the increase in observed outliers, as well as the increase in skew for distributions in the tail ends of the range of recovered conserved single-copy genes. Download FIG S3, PDF file, 0.1 MB. Copyright © 2017 Landry et al. 2017 Landry et al. This content is distributed under the terms of the Creative Commons Attribution 4.0 International license.

**TABLE 1:** Genome completion, estimated genome size, and metadata for 5 SAR202 SAGs^a^

Site	Depth (m)	Group	SAG name	No. of identified conserved genes	Estimated fraction complete	Estimated genome size (bp)
HOTS	770	3	AAA240-O15	33	0.47	3,032,068 ± 289,181
	770	3	AAA240-N13	20	0.33	4,259,313 ± 234,134
South Atlantic	800	3	AAA001-F05	9	0.19	6,949,189 ± 161,421
	800	3	AAA007-M09	3	0.08	13,153,458 ± 88,969
North Atlantic	511	5	AB-629-P13	42	0.56	1,436,137 ± 158,466

^a^ Completion and size estimates are based on a set of 71 core genes found to be conserved in all *Chloroflexi* included in our analysis.

### Environmental distribution.

Reciprocal best-BLAST recruitment of SAR202 SAGs against the Microbial Oceanography of ChemolithoAutotrophic planktonic Communities (MOCA) and HOTS depth profiles are shown in Fig. S4 and S5 and Table S2 in the supplemental material. Average nucleotide identities (ANIs) to bathypelagic metagenomic data were very high (≥90%), and the genomes were well represented in samples from 4,000 to 5,000 m of depth. Although this survey was limited to the few deep-ocean metagenomes that were available, the results indicate that the group III SAGs, which were isolated from depths of 770 to 800 m, likely represent abundant bathypelagic cells (14). SAR202 relative recruitment was highest in the deepest samples (3.8% [Table S2]), but the real abundance of SAR202 DNA is expected to be significantly higher since, in proportion to incompleteness, SAG recruitment underestimates genome abundance. The group V SAR202 SAG, which was isolated from a depth of 511 m in the North Atlantic, contributed only 196 reads out of the 20,152 recruited reads from MOCA and 115 out of 12,293 reads from the HOTS depth profile. Therefore, the environmental relevance of this subclade in the open ocean remains unknown.

10.1128/mBio.00413-17.5 FIG S4  Reciprocal best-BLAST recruitment of SAR202 contigs to the MOCA North Atlantic depth profile. Depths of recruited fragments are color coded according to the key in the upper right. Download FIG S4, PDF file, 0.3 MB. Copyright © 2017 Landry et al. 2017 Landry et al. This content is distributed under the terms of the Creative Commons Attribution 4.0 International license.

10.1128/mBio.00413-17.6 FIG S5  Reciprocal best-BLAST recruitment of SAR202 contigs to the HOTS/ALOHA depth profile. Depths of recruited fragments are color coded according to the key in the upper right. Download FIG S5, PDF file, 0.2 MB. Copyright © 2017 Landry et al. 2017 Landry et al. This content is distributed under the terms of the Creative Commons Attribution 4.0 International license.

10.1128/mBio.00413-17.9 TABLE S2  Reciprocal best-BLAST recruitment of SAR202 single-amplified genomes against reads from two metagenomic depth profiles. Note that the highest levels of recruitment are from the deepest samples, indicating that these cells likely interact with chemical species found in the most remote depths of the ocean. Download TABLE S2, PDF file, 0.1 MB. Copyright © 2017 Landry et al. 2017 Landry et al. This content is distributed under the terms of the Creative Commons Attribution 4.0 International license.

### Global phylogeny of FMN/F420-dependent monooxygenases.

The most salient feature of the SAR202 genomes was a high number of paralogous protein families, which was apparent in comparisons with other marine bacterial species (Table 2; see Fig. S6 in the supplemental material). Proliferations of paralogs often accompany the evolutionary expansion of organisms into new niches, wherein the paralogs provide the new functions that are needed to adapt (43, 44). We focused on the paralogs for clues that would help us understand what early events in evolution might have propelled SAR202 to its highly successful colonization of the dark-ocean habitat.

10.1128/mBio.00413-17.7 FIG S6  The fraction of protein-coding genes in the SAR202 genomes with discernible paralogs at a number of similarity thresholds compared with *Ktedonobacter racemifer* and a number of other representative marine bacteria. The SAR202 genomes and *Ktedonobacter* show consistently higher levels of paralogs than the other organisms. Download FIG S6, PDF file, 0.1 MB. Copyright © 2017 Landry et al. 2017 Landry et al. This content is distributed under the terms of the Creative Commons Attribution 4.0 International license.

**TABLE 2:**
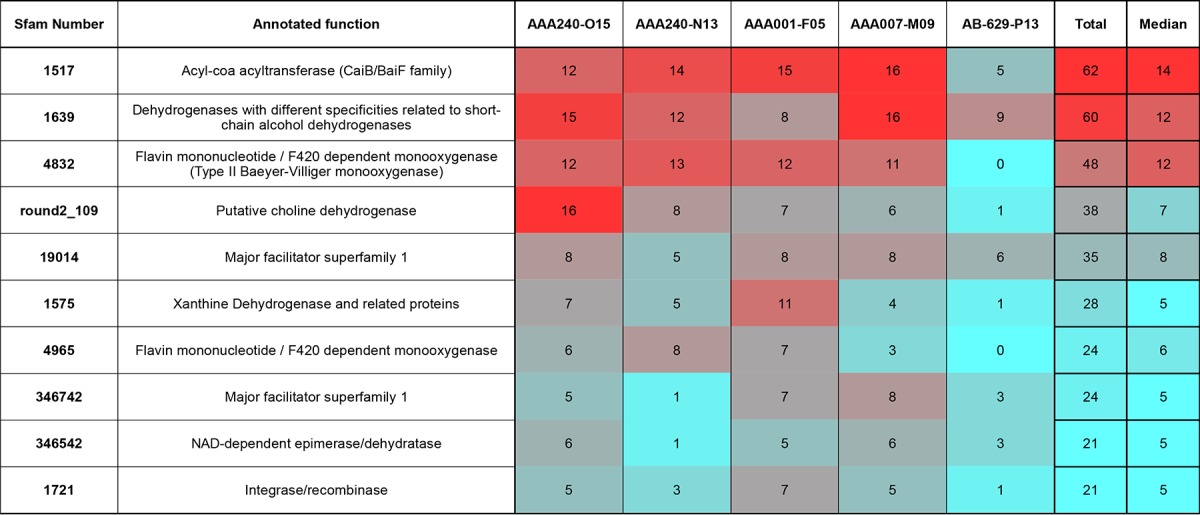
The 10 most highly represented Sfam protein families from SAR202 SAG assemblies^a^

^a^ The 10 families shown here represent a number of paralogous protein sequences. This table shows remarkably high representation of some families, and there are a number of additional Sfam families present in the genomes with identical structural and functional annotations.

**FIG 2:**
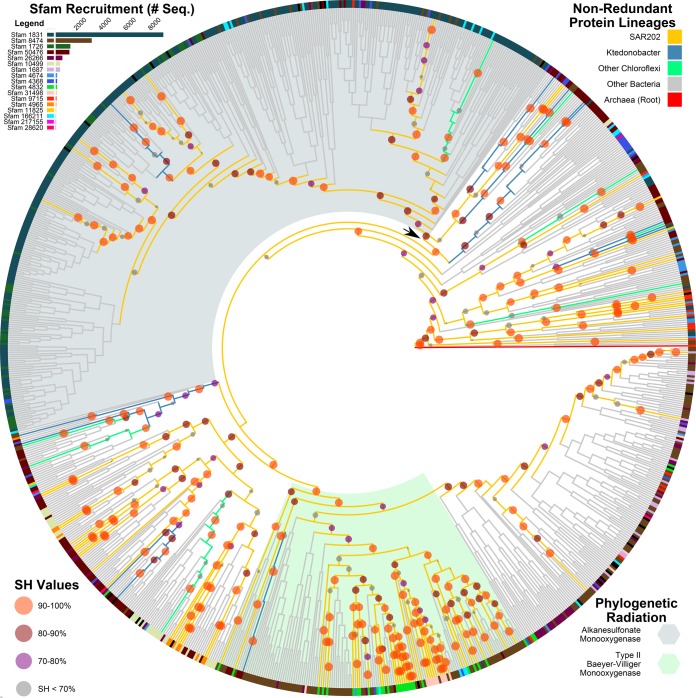
Global phylogeny of bacterial FMNO proteins rooted on a consensus sequence from archaeal FMNO homologs. This tree shows that SAR202 FMNO paralogs branch deeply in trees, indicating ancient diversification of these genes in SAR202 and related *Chloroflexi*. Ancestry of SAR202, *Ktedonobacter*, and other *Chloroflexi* are indicated in descending priority by the color of tree segments according to the key in the upper right. Shimodaira-Hasegawa test values are represented by circles on the internal nodes of SAR202 and *Chloroflexi*, with radii proportional to confidence and coloring according to the key in the lower left. Terminal nodes are color coded by majority Sfam family; a color key and number of sequences recruited from databases (Swiss-Prot and RefSeq) for each family are shown in the upper left-hand corner. Radiations associated with enzyme functions are highlighted according to the key in the lower right. The majority of SAR202 sequences are in the radiation associated with Type II Baeyer-Villiger monooxygenase (BVMO) functionality. The arrow indicates the deep node at which BVMO enzymes diverged from the more common alkanesulfonate monooxygenases.

Prominent among the paralogs were 152 sequences representing a large and highly diverse family of flavin mononucleotide(FM)/F420-dependent monooxygenase catalytic subunits (FMNOs) (45–47). hmmscan (48) with Sfams revealed that the five SAR202 genomes altogether harbored 48 genes related to Sfam 4832, which is closely related to the Sfam 8474 FMNO family of Baeyer-Villiger monooxygenases (BVMOs). An additional 104 sequences from the genomes were associated with 16 related Sfam models in the FMNO family. These 17 models were used to recruit 15,623 amino acid sequences from the Refseq65 bacterial protein set, as well as an additional 115 amino acid sequences from Swiss-Prot/UniProtKB. Dereplication of these sequences yielded consensus sequences for 990 gene lineages. Figure 2 is an approximate maximum likelihood tree made from the final FMNO alignment. SAR202 FMNOs spanned the full range of the FMNO tree, often branching with other *Chloroflexi-*derived sequences.

FMNOs and other flavoenzymes have been classified into 8 classes (A to H) in the system devised by van Berkel (45–47): they catalyze a variety of oxidation reactions on an enormous range of substrates, including aromatic ring hydroxylation, desulfurization, alkane oxidation, Baeyer-Villiger oxidation, epoxidation, and halogenation. Predominant annotations for protein sequences recruited by the 17 FMNO Sfam hidden Markov models (HMMs) were “alkanesulfonate monooxygenase,” “hypothetical protein,” “monooxygenase,” “luciferase,” “*N*5,*N*10-methylene tetrahydromethanopterin reductase and related flavin-dependent oxidoreductases,” or “alkanal monooxygenase.” All of these would be categorized as class C flavin-dependent monooxygenases in the nomenclature system for flavoenzymes devised by van Berkel (45–47). Bacterial luciferase is the canonical class C monooxygenase that oxidizes aldehydes to carboxylic acids. Alkanesulfonate monooxygenases (SsuD) are also well represented among class C FMNOs; they catalyze the cleavage of carbon-sulfur bonds in a wide range of sulfonated alkanes and have high affinities for sulfonated compounds with large conjugated R-groups (49). Additional class C monooxygenases include nitrilotriacetic acid monooxygenase, dibenzothiophene monooxygenase, and type II Baeyer-Villiger monooxygenases (46).

The most common annotated function of FMNO sequences recruited from public databases is “alkanesulfonate monooxygenase,” but this functional annotation is largely associated with one protein family, Sfam 1831. While prevalent across the tree of life, this family is only represented by a single protein from SAR202. Sfam 1831 represented 296 nonredundant lineages and 8,695 of the recruited FMNO protein sequences, while the other 16 models were assigned to 694 of the lineages representing 7,195 amino acid sequences. Most of the *Chloroflexi* FMNO lineages are phylogenetically diverged from Sfam 1831 (Fig. 2). While alkanesulfonate monooxygenases are structurally and phylogenetically related to the other FMNOs, they diverge at a deep-tree node, and most of the FMNO diversity represented by SAR202, *Ktedonobacter* and other *Chloroflexi*, is on the other side of this node, among the non-alkanesulfonate monooxygenase lineages.

**FIG 3:**
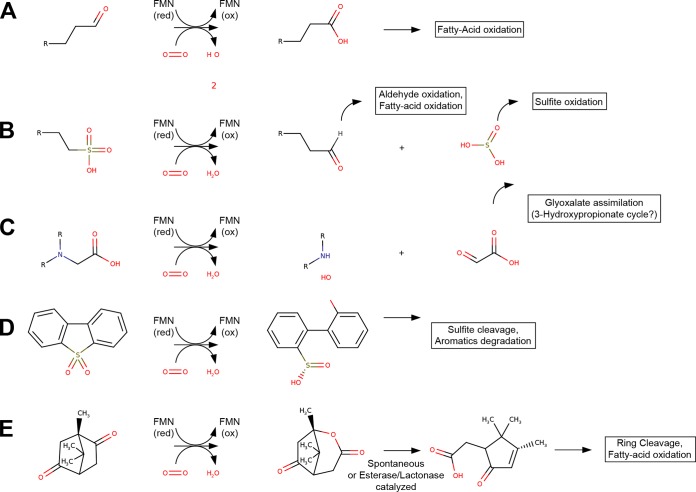
Types of reactions catalyzed by group C flavin monooxygenase proteins and possible fates of reaction products (in boxes). Canonical reactions for enzymes in this protein family include (A) alkanal monooxygenases, which convert aldehydes to fatty acids; (B) alkanesulfonate monooxygenases, which catalyze the cleavage of carbon-sulfur bonds to produce an aldehyde and a free sulfite; (C) nitrilotriacetate monooxygenase, which catalyzes the removal of a glyoxalate group from an aminocarboxylic acid; (D) dibenzothiophenone monooxygenase, which catalyzes the cleavage of a carbon-sulfur bond to produce a substituted biphenyl; and finally (E) the type II Baeyer-Villager monooxygenases, which catalyze the insertion of a single oxygen adjacent to a ketone or aldehyde group. The resulting lactone is decyclized spontaneously or through an esterase-catalyzed reaction. red, reduced; ox, oxidized.

Genome annotation predicts that the majority of SAR202 FMNO enzymes are Baeyer-Villiger monooxygenases that break alicyclic rings by an oxidative mechanism. Interpreting these genes in the context of other genes found in SAR202 genomes leads us to propose that the function of the SAR202 BVMOs is to activate recalcitrant molecules for biodegradation. Closer examination of the Sfam 4832 family sequences (which contain the most sequences of the FMNO families represented in SAR202 [Table 2]) using the PHYRE2 structural homology recognition server (50) showed high predicted structural similarity of these proteins to 3,6-diketocamphene 1,6-monooxygenase (template/fold identification c2wgkA). 3,6-Diketocamphene 1,6-monooxygenase is a type II Baeyer-Villiger monooxygenase that catalyzes the insertion of an oxygen atom into the alicyclic 3,6-diketocamphene molecule to convert a ketone group to an ester (Fig. 3). In the camphor degradation pathway, this insertion is followed by the spontaneous decomposition of the unstable ring structure into a partially linearized carboxylic acid (51). Additional support for type II BVMO functionality is found in our phylogenetic data. The majority of the Sfam 4832 nonredundant lineages (for which there are no representative proteins in Swiss-Prot) share an ancestry with the Sfam 8474 lineages, branching together at a single deep node in the phylogenetic analysis (Fig. 2). Sfam 4832 is uncommon across the bacterial tree, with only 64 other proteins recruiting to this family from the entire RefSeq database of bacterial genomes (mostly from a number of *Actinobacteria*) and none from Swiss-Prot. The only sequences recruited to the closely related Sfam 8474 HMM from the manually curated Swiss-Prot database, however, were the type II BVMO enzymes 2,5-diketocamphene 1,2-monooxygenase and 3,6-diketocamphene 1,6-monooxygenase of *Pseudomonas putida* (51) and the limonene 1,2-monooxygenase of *Rhodococcus erythropolis*, which catalyzes the epoxidation of an alicyclic ring structure. While the most common type of FMNO found in the SAR202 genomes is these putative type II Baeyer-Villiger monooxygenases, divergent SAR202 sequences in the FMNO family may perform other canonical catalytic functions of class C FMNOs, such as the removal of sulfur and nitrogen groups from alkanesulfonates, or aminocarboxylic acids, respectively, yielding alkanes for assimilation or oxidation. The presence of a sulfite oxidase suggests that sulfite derived from organosulfur compounds might be used as an energy source; alternatively this enzyme might benefit cells by detoxifying organosulfur-derived sulfites. Alternatively, the presence of an alkanesulfonate monooxygenase may simply impart the ability to utilize organic sulfur to fulfill the cell’s sulfur requirements.

Sequence diversity within the SAR202 FMNOs and the pattern of shared deep branches with *Ktedonobacter* FMNOs cannot be explained by horizontal gene transfer without accepting a remarkable amount of accidental coincidence. Rather, this pattern strongly suggests the early expansion of these paralogs in the common ancestor of *Ktedonobacter* and SAR202. In the absence of empirical experimental data, the substrates for these divergent type II BVMOs cannot be predicted with certainty; however, their annotations were consistent with predictions of alkane degradation functions elsewhere in the genomes, prompting us to conclude that the SAR202 FMNOs are most likely involved in the activation of long-chain or cyclic aliphatic molecules for degradation. The ancient origin of the SAR202 clade, its deep-branching diversity, and its current role in ocean ecology are compatible with the hypothesis that these FMNOs diversified in a progenitor of SAR202 that expanded into a niche that required the strong oxidative activity of FMNOs.

### Acyl-CoA:CoA transferases.

In addition to the monooxygenase sequences, a number of other paralogous protein families were encoded in the SAR202 genomes. The most prominent of these are a family of acyl coenzyme A (acyl-CoA):CoA transferases (formerly bile acid-inducible protein f; Caib/Baif [Sfams 1517, 17645, 25993, 33148, 42237, and 67858]). The Baif form of this enzyme is thought to catalyze the release of deoxycholate, a sterol derivative, with the subsequent binding of cholate to CoA, and has been shown to be active over a wide range of substrates (52). The major advantage of these proteins may be that they allow the integration of new substrate into degradation pathways without the consumption of reductant or ATP. A subset of the putative acyl-CoA transferases encoded by SAR202 have top hits to the DddD enzyme of *Marinomonas* (UniProtKB, SP no. A6W2K8.1; NCBI GI no. 928589252) compared to the Swiss-Prot/UniProtKB database. The DddD of *Marinomonas* is a dimethylsulfoniopropionate (DMSP) transferase/lyase that has been shown to be homologous to Caib/Baif-type proteins, indicating the possibility of SAR202 using the common algal osmolyte DMSP, although DMSP as a substrate is unlikely to be present in the deep ocean (53). The lyase activity of the DddD protein also prompts us to believe that it is possible that some of these CoA transferase proteins may also encode novel lyase functions for additional substrates.

### Short-chain dehydrogenases.

The five SAR202 genomes altogether contained 60 proteins belonging to Sfam 1639 (Table 2), most of which are annotated as “oxidoreductase” or “short-chain dehydrogenase.” In comparisons to the Swiss-Prot/UniProtKB database, a number of these enzymes had high amino acid identities (AAIs [~50%]) to cyclopentanol dehydrogenase or 3-α (or 20-β)-hydroxysteroid dehydrogenase. There is a profound similarity between the reactions catalyzed by these enzymes. Cyclopentanol dehydrogenase and 3-α (or 20-β)-hydroxysteroid dehydrogenase, while active on seemingly different substrates, both convert an alicyclic-bound alcohol group to a ketone, producing reduced NADH as a product (54–56). The conversion of sterols to ketones is illustrated by the reaction catalyzed by 3-α (or 20-β)-hydroxysteroid dehydrogenase. The production of similar enzymes by SAR202 would help explain the documented vertical pattern of sterols and steroid ketones throughout the meso- and bathypelagic ocean (57, 58). The conversion of alcohol groups to ketones is a priming mechanism for subsequent BVMO oxidation (59). Given the large number of putative Baeyer-Villiger monooxygenases in SAR202 genomes, we propose that SAR202 short-chain dehydrogenase paralogs prepare deep-ocean DOM species for oxygen insertion and ring opening through a lactone intermediate, partially linearizing the molecule and allowing the conversion of a recalcitrant alicyclic ring structure to a more labile carboxylic acid.

The gene annotations described above lead us to hypothesize that SAR202 might participate in the oxidation of a variety of cyclic alkanes, including sterol and hopanoid-like structures. Sterols and hopanoids are chemically related triterpenoid molecules that play analogous roles in the cell membranes of eukaryotes and bacteria, respectively (27), and have been shown to be common in the water column (29, 60–64). These molecules exhibit highest concentrations in the lower euphotic zone/upper mesopelagic and decline with depth. There is a corresponding appearance and subsequent slow disappearance of steroid ketone molecules with depth that is hypothesized to be the result of biotic conversion of sterols to steroid ketones (57, 58, 65, 66). These patterns are consistent with the previously reported vertical range of SAR202 (Fig. S4 and S5) throughout the mesopelagic (10, 14).

### Transporter proteins.

Across the five genomes, there were 129 proteins annotated as subunits of major facilitator superfamily 1 (MFS1) transporters. In comparisons to the Swiss-Prot/UniProtKB database, these proteins were most similar to proteins annotated as multidrug efflux transporters. MFS1 transport proteins are solute antiporters that have in the past been reported to be widely promiscuous in substrate, with individual enzymes capable of transporting a wide range of compounds in and out of the cell (67–69). The genomes also encoded 14 proteins (from Sfams 1548, 13968, 18128, 50865, and 54520) that encode tripartite ATP-independent periplasmic (TRAP) transporters. TRAP transporters are associated with a wide variety of compounds, including dicarboxylic acids and sugars (70). The five genomes additionally included a total of 182 proteins involved in ABC transport systems with affinities for a variety of substrates, including putative transporters for inorganic nitrogen and phosphate species as well as organic phosphonates.

### Other oxidative enzymes.

Each of the genomes contained several versions of the promiscuous enzyme cytochrome P450, which catalyzes the formation of hydroxyl, carboxyl, or carbonyl groups from alkanes, including enzymes of the CYP125 type that has been previously implicated in sterol degradation (71). Also present were most enzymes for the oxoglutarate:ferredoxin oxidoreductase variant of the tricarboxylic acid (TCA) cycle, which oxidizes acetyl-CoA, yielding reduced ferredoxin. Bacterial cytochrome P450 enzymes are typically activated with reduced ferredoxin, which is a more electronegative carrier than the more common nucleotide cofactors NADH and NADPH. These synergistic genome features are further evidence of a metabolism equipped for the oxidation of a broad range of heterogeneous recalcitrant compounds.

The assemblies also contained multiple families annotated as carbon monoxide dehydrogenase/xanthine dehydrogenase/aldehyde oxidases, including a total of 28 proteins associated with Sfam 1575. Some members of the *Ktedonbacteria* have been shown to be capable of oxidizing carbon monoxide (32), and low concentrations of carbon monoxide present (<1 nM) in the deepwater column, indicate that there may be a CO sink in the meso- and bathypelagic (72). However, given the other functions of enzymes in this protein family and the diverse alkane degradation genes contained in our assemblies, it seems more likely that many or most of these proteins have functions similar to those of xanthine dehydrogenase rather than carbon monoxide dehydrogenase. Xanthine dehydrogenase enzymes often additionally show a xanthine oxidase activity (73), and some versions of this enzyme are active across broad substrate ranges (73, 74). We speculate these enzymes could be involved in the initial steps of degrading complex substrates to more tractable forms by the addition of hydroxyl groups to alkanes or the formation of ketones from alkene groups. Either of these reactions would initiate priming of an alkanal molecule for eventual oxygen insertion by a Baeyer-Villiger monooxygenase.

### Choline degradation and C_1_ oxidation.

The SAR202 genomes contained a large number of proteins related to choline dehydrogenases that likely have analogous functions, such as oxidizing alcohols to aldehydes (Sfams 108560, 1557, 163373, 2157, 27990, 38781, and round2_109). Sfam family round2_109 alone recruited 38 proteins from the five genomes. The SAR202 genomes also harbored a number of the other proteins that have predicted functions in the choline degradation pathway, including most of the subunits of multimeric sarcosine oxidase (EC 1.5.3.1) and serine hydroxymethyltransferase (EC 2.1.2.1). The genomes additionally included genes for C_1_ (one-carbon) oxidation with components of the tetrahydrofolate-linked demethylation pathway and genes for formaldehyde oxidation (EC 1.2.1.46) and formate dehydrogenase (EC 1.2.1.2), which oxidizes formate to CO_2_. This suite of genes likely confers upon these cells the ability to demethylate compounds such as choline and to oxidize the methyl groups to CO_2_ in an energy-yielding reaction. The presence of a multimeric sarcosine oxidase as well as a DMSP lyase in some of the SAR202 genomes also indicates that it can metabolize some low-molecular-weight, semilabile forms of carbon. If SAR202 does indeed utilize these smaller forms of organic carbon, it may contribute to a “priming effect” in some deepwater systems where refractory DOM is readily degraded when labile carbon is available to support baseline metabolism (5, 75, 76).

### 3-Hydroxypropionate cycle.

The group III SAR202 genomes contained genes predicted to encode many enzymes of the 3-hydroxypropionate cycle (3HPC), including subunits of the key trifunctional malyl/methylmalyl/citramalyl lyase (see Table S3 in the supplemental material) (77, 78). The 3-hydroxypropionate cycle was first described in the *Chloroflexi* phylum member *Chloroflexus aurantiacus*, where it was interpreted as a pathway of autotrophy in the absence of conventional carbon fixation pathways (77, 78). While associated with carbon fixation, in the first experimental papers describing the cycle and its biochemical role, the authors stated that it likely is repurposed for this cause and that the original role of the 3-hydroxypropionate cycle was probably to facilitate the utilization and salvage of a wide range of carbon substrates (78, 79). The SAR202 genomes harbored propionyl/acetyl-CoA carboxylases that are associated with the CO_2_ fixation steps of the cycle, as well as subunits of a key enzyme, trifunctional malyl/methylmalyl/citramalyl lyase, that is associated with the full bi-cycle (identified by Phyre2 structural prediction server, 100% predicted structural similarity to the trifunctional lyase of *Chloroflexus aurantiacus*). Due to the low estimated completeness of the genomes, we cannot rule out the possibility that the cycle plays a chemolithotrophic role in SAR202, but the lack of coding sequences definitively associated with the chemolithotrophic metabolism and the overwhelming presence of genes that appear to be involved in complex alkane degradation lead us to hypothesize that the primary role of the 3HP cycle in SAR202 is the assimilation of metabolic intermediates that result from the catabolism of complex deep-ocean DOM.

10.1128/mBio.00413-17.10 TABLE S3  Candidate protein sequences found within the SAR202 genomes that potentially code for enzymes or enzyme subunits required by the 3-hydroxypropionate cycle. Download TABLE S3, PDF file, 0.1 MB. Copyright © 2017 Landry et al. 2017 Landry et al. This content is distributed under the terms of the Creative Commons Attribution 4.0 International license.

We propose that a full or partial version of this pathway may be used in SAR202 to facilitate absorption of varied metabolic intermediates produced by the degradation of recalcitrant DOM. Most 3HPC intermediates are short 3- to 4-carbon carboxylates or dicarboxylic acids, representative of the types of compounds that would remain following incomplete oxidation of modified, complex, or branched-chain fatty acid oxidation. Autotrophically grown *Chloroflexus aurantiacus* cells have also been shown to preferentially absorb organic carbon through some of these intermediate forms, despite an excess of CO_2_ and H_2_ available for carbon fixation (79). Segments of the 3HPC, involving one or more successive enzymes, are implemented in the metabolism of other marine microbes to assimilate compounds such as short carboxylic acids and aldehydes produced by catabolic pathways, such as in DMSP catabolism (79, 80).

### Fatty acid degradation.

Gene candidates for all of the central enzymes involved in fatty acid beta-oxidation were found among the five SAR202 genomes. All three genes of the propionyl-CoA pathway (overlapping with the 3-hydroxypropionate cycle) for odd-length and methylated fatty acid degradation were found in multiple members of the SAR202 genomes. This set includes propionyl-CoA carboxylase (EC 6.1.4.3), methylmalonyl-CoA epimerase (EC 5.1.99.1), and methylmalonyl-CoA mutase (EC 5.4.99.2). The presence of a full beta-oxidation pathway, and a propionyl-CoA degradation pathway, indicates that SAR202 is able to fully degrade both odd- and even-chain fatty acids.

Putative genes for alpha-oxidation of branched alkanes, which are highly unusual among bacteria, were found among the genomes. Both phytanoyl-CoA dioxygenase and 2-hydroxyphytanoyl-CoA lyase were present. These genes indicate that SAR202 may be capable of degrading branched-chain alkanes that are resistant to beta-oxidation, including phytanyl compounds, such as the phytol side chains of chlorophyll molecules and the isoprenyl chains of archaeal lipids.

### Aromatic degradation.

A pathway for catabolism of aromatic compounds could not be completely reconstructed, but the SAR202 SAGs harbored multiple genes encoding enzymes involved in aromatic carbon catabolism, including proteins with predicted functions in 4-hydroxyphenylacetate degradation, protocatechuate degradation, catechol degradation, and phenylpropionate degradation. Also present were ring-hydroxylating and ring-cleavage dioxygenases and isomerases that could be involved in degradation of aromatics. The diversity and prevalence of these genes lead us to propose that SAR202 cells are able to catabolize some aromatic compounds.

### Carboxylate degradation.

Genes encoding proteins involved in carboxylic acid degradation pathways were abundant in the SAR202 genomes. Pathways for acetoacetate degradation and 2-oxobutanoate degradation were complete or nearly complete, and there were a large variety and number of acyl-CoA or formyl-CoA transferase homologs: in addition to the 62 aforementioned Caib/Baif-type proteins in Sfam 1517, there were 11 proteins from Sfam families 172716, 17645, 25993, 33148, 42237, and 67858. Coenzyme A can serve as a carrier for a wide variety of acyl compounds during biological oxidation, and we interpret the proliferation of CoA transferases in these cells as evidence they might be capable of metabolizing many different acyl compounds by forming CoA thioesters. Such acyl compounds are among the products predicted to form through the action of the strongly oxidative FMNO and P450 enzymes found elsewhere in the genome. The ability of acyl-CoA transferases to remove substrates that are easily oxidized without a carrier (such as formate) from CoA cofactor through substitution of a new ligand without the use of ATP is an energetically efficient mechanism used by cells to move substrates through oxidative pathways. This strategy seems well suited to the niche we propose for SAR202 of oxidizing some of biology’s least favorable organic substrates in the dark ocean.

### Sialic acid.

Acetylated aminosugars, including sialic acid, have been shown to be a major component of marine DOM, accounting for up to 17 to 43% of high-molecular-weight DOM at depth, making them a large component of the heteropolysaccharide portion of deep-ocean DOM (5, 27, 81, 82). Sialic acids often decorate glycoproteins and are abundant in S-layers, which are proteinaceous outer wall structures found in many bacteria and archaea. SAR202 genomes have multiple genes predicted to function in sialic acid biosynthesis. These include *N*-acetylneuraminate synthase, CMP-*N*-acetylneuraminate synthase, and candidates for polysaccharide biosynthesis protein CapD. While the existence of an S-layer has not been documented in cultured *Chloroflexi*, S-layers have been predicted for members of the phylum due to the presence of sialic acid biosynthetic enzymes observed previously in single-amplified genomes and assembled metagenomes (40, 83). The presence of sialic acid biosynthetic pathways has ecological implications: S-layers provide protection against phage predation in bacteria (84–87), presumably by modifying or masking cell surface epitopes. These observations raise the possibility SAR202 S-layers might contribute to the high-molecular-weight fraction of deepwater DOM, through a mechanism consistent with ideas expressed by the microbial carbon pump hypothesis.

### Metabolic overview.

**FIG 4:**
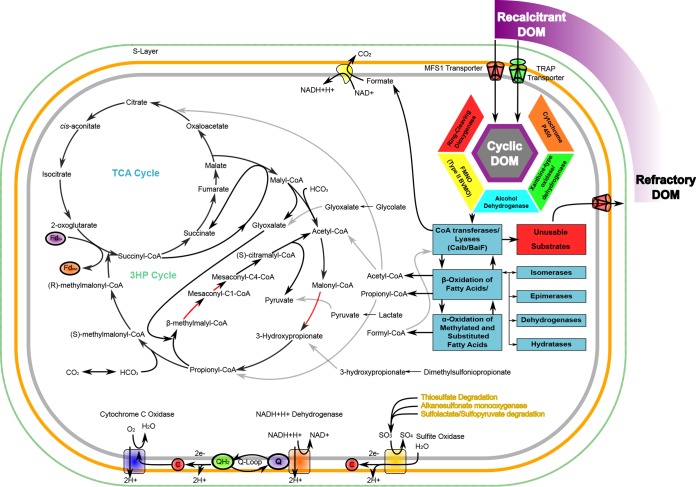
A proposed metabolic schema for cells of the SAR202 clade. Oxidative enzymes encoded in the SAR202 genomes provide semispecific initial oxidation of heterogenous recalcitrant DOM on the cell surface or upon initial transport of these compounds into the cell. Carboxylate degradation products are ligated into a pool of CoA-linked substrates via acyl-CoA transferases. CoA-linked substrates move to the beta- and possibly alpha-oxidation pathways. Partial degradation products of fatty acids and substituted alkanes released from the partial oxidation of recalcitrant DOM may provide intermediates for absorption via the 3-hydroxypropionate cycle (3HPC). Incompletely degraded substrates may be substituted for more active substrates using acyl-CoA transferase activity, with released products being exported from the cell via a number of MFS1 transport proteins with documented promiscuous transport activities, thus contributing to a more refractory environmental pool of marine dissolved organic matter. 3HPC pathway gaps for which no suitable gene candidates were found in the draft genomes are marked in red. While it is uncertain as to whether the enzymes of the 3HPC shown here participate in carbon fixation, even an incomplete 3HPC could provide a means of assimilating a number of small carbon intermediates into central metabolism. Several mechanisms for cleavage of sulfite from sulfur and organosulfur compounds are encoded by the genome and may provide a source of sulfur for growth or a substrate for sulfite oxidase.

A metabolic reconstruction based on annotated SAR202 gene functions is shown in Fig. 4. The gene content and predicted metabolism of SAR202 genomes are distinct from any described previously and ostensibly well suited to the niche of oxidizing recalcitrant DOM in the deep ocean. The key to reconstructing this metabolism is the presence of multiple, anciently diverged families of paralogous enzymes, particularly the FMNOs, which are united by their strongly oxidative catalytic mechanism. In a general sense, the reaction mechanisms of these enzymes are especially suited to the oxidation of recalcitrant compounds and produce labile alkanyl intermediates that are favorable carbon or energy sources, but could be highly diverse, depending on the heterogeneity of the parent compounds. From a thermodynamic perspective, it seems likely that these compounds are recalcitrant because a powerful oxidation step is required to transform them into molecules that are tractable for further catabolism. The derivatives of the oxidation step are alkanal molecules that are potentially rich sources of energy. In the scenario we propose, paralogs diversified in response to evolutionary pressure to use a variety of substrates via a common pathway mechanism. Other enzymes likely contributing to the initial oxidation of marine DOM include cytochrome P450, short-chain alcohol dehydrogenases, enzymes associated with aromatic degradation, and purine dehydrogenases/oxidases.

**FIG 5:**
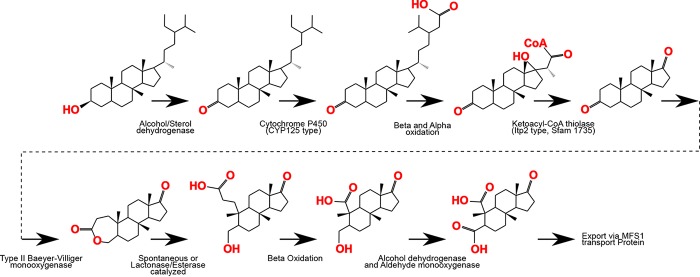
A possible alicyclic carbon degradation mechanism, with stigmastanol as an example, using reactions thought to be encoded in the SAR202 genome annotations. The side chain is activated through a CYP125 cytochrome P450. This is followed by beta- and alpha-oxidation of the side chain. The sterol is converted to a steroid ketone through the activity of a dehydrogenase, and a type II Baeyer-Villiger monooxygenase subsequently creates a lactone intermediate. The lactone ring can decompose spontaneously or through a lactonase- or esterase-catalyzed reaction. Alcohols can be oxidized to aldehydes through the action of alcohol dehydrogenases. Aldehydes can be further converted to carboxylic acids through the action of an aldehyde monooxygenase. Exposed carboxylic acid residues can be further degraded through beta-oxidation, leaving a carboxyl- and carbonyl-rich alicyclic fragment with limited opportunities for further degradation.

To illustrate, Fig. 5 uses predicted enzyme functions from the SAR202 genomes in a hypothetical pathway for degradation of the common phytoplankton lipid stigmastanol. Through a combination of the activities of cytochrome P450, alpha- and beta-oxidative enzymes, alcohol dehydrogenase, and a Baeyer-Villiger monooxygenase, reactive moieties, such as the side chain, can be removed and rings broken by the insertion of oxygen atoms, which results in decyclization through a lactone intermediate. Further degradation results in a final product resembling deep-sea DOM structures proposed by Hertkorn et al. (27). This is not to say that SAR202 is degrading sterols/stanols *per se*, but to demonstrate by example that annotated enzymes found within these genomes potentially encode pathways that are capable of partially oxidizing recalcitrant cyclic DOM compounds, resulting in the production of more refractory products. We propose that a variety of small CoA-linked derivatives that result from incomplete oxidation of branched alkanes are assimilated as intermediates via the 3HPC. In this scenario, CoA-linked compounds that are not substrates for the 3HPC assimilation or further oxidation could be eliminated from the CoA pool through the activity of the CoA transferase paralogs that are predicted to function without the consumption of ATP. In the proposed metabolic scheme, these enzymes link the ligation of newly imported or more labile substrates to the displacement of partially oxidized, unusable compounds, providing an entry point for new carbon compounds into degradation pathways, while simultaneously conserving energy.

The reconstructed pathways shown in Fig. 5 include steps that we predict could partially oxidize recalcitrant compounds, but do not explain their complete oxidation to CO_2_. In this reconstruction, we speculate that the multiple MFS1 transport proteins encoded in these genomes function to export substrate fragments that cannot be further oxidized in these cells. If this scenario is correct, then it seems plausible that group III SAR202 contributes to the accumulation of refractory DOM in the deep ocean through the exudation of partially degraded substrates that are resistant to further catabolism. Such a scenario would place SAR202 in a key position in the marine carbon cycle, catalyzing steps in the conversion of recalcitrant DOM to refractory compounds by partially oxidizing recalcitrant compounds and further lowering their chemical reactivity. Similar scenarios have been proposed for freshwater DOM (88).

### Conclusions.

This study of SAR202 genomes provided unexpected insights into the metabolic strategies of these deep-ocean bacteria. In these genomes, we discovered genes for an unusually rich assortment of enzymes implicated in the oxidation of recalcitrant organic compounds. These genes include multiple FMNO paralogs, P450, and enzymes predicted to function in sterol and aromatic compound catabolism. Due to the deep branching and high diversity of the SAR202 clade, as well as recent preliminary descriptions of SAGs from other subclades of SAR202 (89), it is probable that a number of these features are specific to the group III SAR202, and it is expected that there will be significant differences between the group III SAR202 and the other subclades. We also found in these genomes a key evolutionary signature of an ancient proliferation of FMNOs. Tree topologies place this event near the root of the *Chloroflexi*, possibly in a common ancestor of SAR202 and *Ktedonbacter*, who share a basal position in the phylum in our phylogenomic analysis. These results suggest an ancient origin of this metabolism.

The findings we present and the current successful occupation of the dark-ocean niche by SAR202 lead us to hypothesize that SAR202 diverged soon after the atmosphere became oxidized and expanded into the niche of oxidizing semilabile and recalcitrant DOM by cleavage with powerful BVMOs and other oxidative enzymes. Kim et al. (90) demonstrated that a diversification of oxygen-consuming enzyme families occurred between 1.4 and 2.9 billion years ago, roughly coincident with the rise in atmospheric oxygen and the period through which oxygenation of the prehistoric oceans progressed (91). Their analysis also indicated that enzymatic reactions involving sulfonates only appeared following the initial increase in oxygen (90), which may help to explain the deep bifurcation between the alkanal monooxygenases associated with SAR202 and the alkanesulfonate monooxygenases in the global phylogeny of these enzymes (Fig. 2). About 2 billion years ago, there was a massive sequestration of carbon into ocean sediment, followed by a correction, both of which are thought to be biologically mediated events (92). A scenario consistent with the observations we report is the expansion of FMNO-dependent catabolism in an ancestor of the SAR202 clade as a part of a larger bacterial proliferation into the ecological niche of consuming marine DOM, in the wake of the slow rise in global oxygen.

The biochemistry of deep-ocean DOM oxidation is largely unexplored, but pioneering laboratory experiments with natural marine communities support the theory that the production of refractory DOM in the deep ocean is biologically mediated, with fresh substrates being transformed into heterogeneous mixtures of recalcitrant DOM in short-term incubations of less than 30 days (28, 93). Experimental approaches such as this provide an avenue for studying biological processes mediated by communities composed of uncultivated cell types. The genome-based hypotheses we report are likely to prove useful for the design of future studies that aim to validate biochemical pathways of deep-ocean DOM oxidation.

We propose the SAR202 group III clade be given the following class and order assignments: *Monstramaria*, classis nov., *Monstramariales*, ord. nov., *Monstramariaceae*, fam. nov. The root of the class, order, and family name stems from the Latin for “sea monster,” to reflect the nearly exclusive presence of members of the SAR202 clade to a number of marine and freshwater environments and represents the ancient divergence of the SAR202 from other *Chloroflexi*, its cryptic genomic and metabolic features, the low amino acid identity of its protein coding sequences to known orthologs, and, most importantly, the great depths in which it resides.

## MATERIALS AND METHODS

### Sample selection, sequencing, assembly, and annotation.

Cell sorting and single-cell genome amplification were performed as described by Swan et al. (94). A full description of sorting, amplification, sequencing, assembly, and annotation is included in Text S1 in the supplemental material. Single-cell sorting, whole-genome amplification, PCR-based analyses, quality control, genome assemblies, and initial genome annotations were performed at the Bigelow Laboratory Single Cell Genomics Center (http://scgc.bigelow.org) (94, 95). A total of five single-amplified genomes (SAGs) belonging to the SAR202 cluster were chosen for whole-genome sequencing and assembly based on multiple displacement amplification (MDA) and quantitative PCR (qPCR) kinetics, phylogenetic affiliation of small subunit rRNA genes, geographic location, and water depth, giving priority to SAGs that were closely related to the SAR202/SAR307 group III SAR202 clones from Giovannoni et al. (10, 12). A combination of Illumina and 454 shotgun sequencing (94) and Illumina-only sequencing (95) were used. Potential contamination and genome completeness were assessed using CheckM (96). Initial functional assignments for individual genes were assigned by the IMG annotation pipeline. Protein sequence annotations were supplemented using the Sifting Families (Sfams) resource of hidden Markov models (97).

10.1128/mBio.00413-17.1 TEXT S1  Supplemental methods. Download TEXT S1, DOC file, 0.1 MB. Copyright © 2017 Landry et al. 2017 Landry et al. This content is distributed under the terms of the Creative Commons Attribution 4.0 International license.

### Phylogenomic inference.

Amino acid sequences from 25 *Chloroflexi* genomes were collected from the IMG database for phylogenomic analysis (see Text S1 for the full list). All five of the SAR202 SAGs were also included: *Chloroflexi* bacterium SCGC AAA001-F05, *Chloroflexi* bacterium SCGC AAA007-M09, *Chloroflexi* bacterium SCGC AAA240-N13, *Chloroflexi* bacterium SCGC AAA240-O15, and *Chloroflexi* bacterium SCGC AB-629-P13. *Pirellula staleyi* DSM 6068 was used as the outgroup. Phylogenomic trees and orthology were determined with a modern reimplementation of the Hal pipeline workflow (98, 99) described previously by Brown et al. (100).

### Genome size estimates.

Genome size estimates were obtained using a set of 71 single-copy conserved core genes derived through a combinatorial core genome analysis of the entire *Chloroflexi* phylum (101–104). *Chloroflexi-*conserved clusters of single-copy genes generated in the first steps of the phylogenomic inference were used as a starting point. To avoid bias, only permanent draft/fully sequenced organisms were used in this analysis. See Text S1 for full details.

### Global phylogeny of FMN/F420-dependent monooxygenases.

The coding sequences of 48 genes with best hits to Sfam 4832 (a “luciferase-like protein”) were extracted. HHSearch (105) was used to recruit 16 additional similar Markov models from the Sfams database. The set of matching Markov models was used with hmmsearch (48) to globally recruit all similar sequences from the manually curated Swiss-Prot/UniProt database (106), as well as all similar bacterial proteins from the RefSeq65 database of protein sequences (107). Single-cell genomes were excluded from this search. The recruited sequences were then reciprocally searched against all Sfam models. The full set of sequences was dereplicated using UCLUST (108) to reduce the original sequence set to representative consensus sequences. Following dereplication, this set of consensus sequences was aligned with MUSCLE (109, 110). Probabilistic masking of the alignment was performed with ZORRO (111). A final tree was created using FastTree (112). Assignment of each of the 990 UCLUST nonredundant lineages to one of these 17 Sfam families was done using a “majority rules” approach, where the number of database sequences recruited by each Sfam model was tallied for each node. Cutoffs and parameters, as well as a detailed description of these analyses can be found in Text S1.

### Reciprocal best-BLAST recruitment.

Genome sequences of all SAR202 SAGs were compared against both the Hawaii Ocean Time Series (HOTS; 22°45′N, 158°00′W) and Microbial Oceanography of ChemolithoAutotrophic planktonic Communities (MOCA; 10°54′N, 44°40′W to 24°30′N, 34°33′W) metagenomic sequences from depth profiles using a reciprocal best-BLAST approach. Scaffolds from each of the five sequenced SAGs were compared with sequences from each of the target depth profiles. Following the initial search, all returned sequences were then reciprocally compared to a copy of the Refseq65 database with single-cell genomes removed and the SAR202 sequences in question added. The highest-scoring hit for each sequence was kept. All hits that matched scaffolds of the SAR202 SAG in question were kept as reciprocal best hits.

### Accession number(s).

Draft genomes of the five SAGs are available in the Integrated Microbial Genomes database under accession no. 2521172610, 2263328036, 2521172608, 2521172609, and 2639762710.
