# Supplementary figures and images for: SAR202 Genomes from the Dark Ocean Predict Pathways for the Oxidation of Recalcitrant Dissolved Organic Matter
Source: mBio. 2017 Apr 18;8(2):e00413-17. doi: 10.1128/mBio.00413-17 (PMC5395668; doi:10.1128/mBio.00413-17)

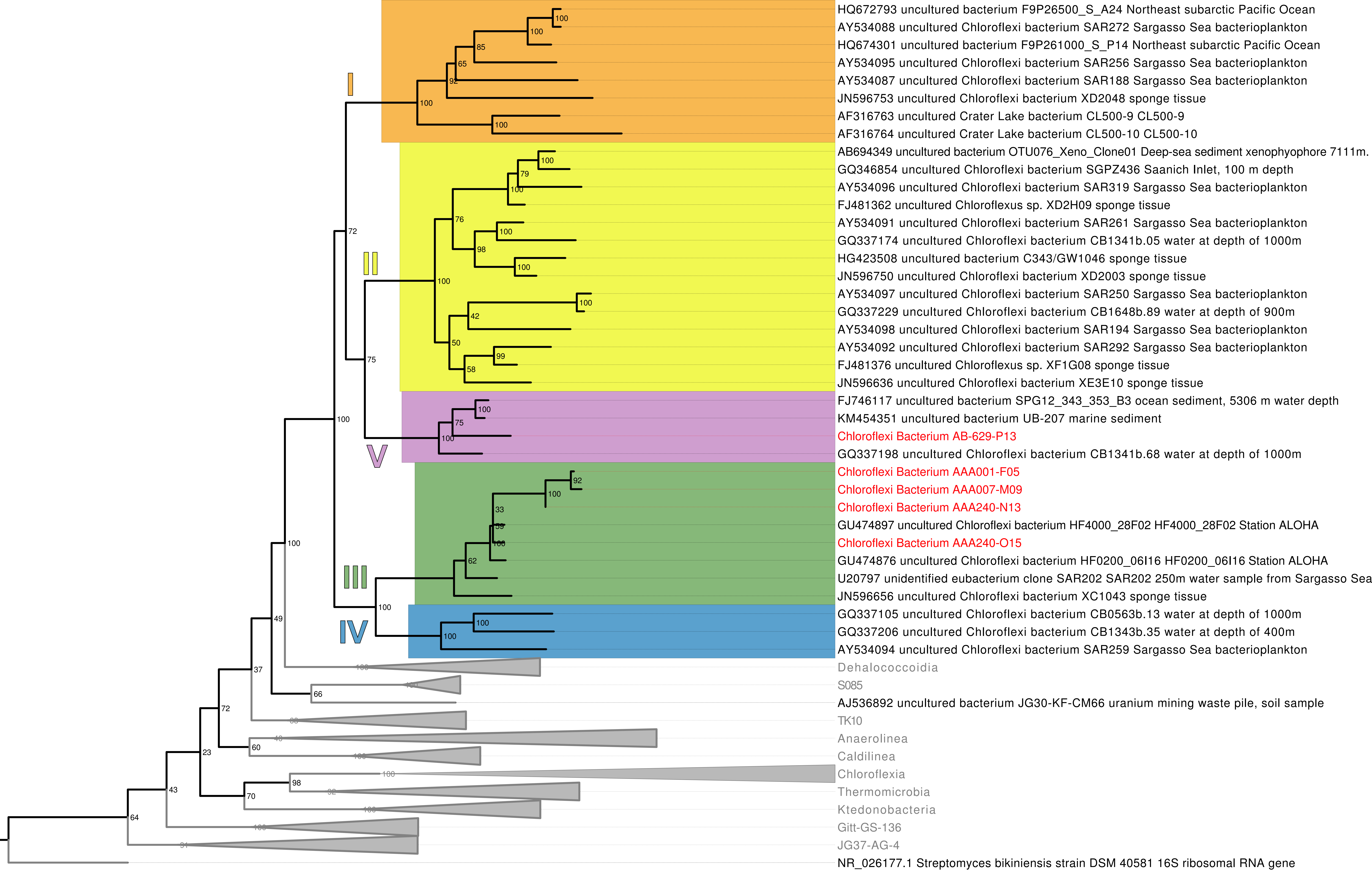

0.2

Supplement: FIG S1 [file mbo002173270sf1.pdf]

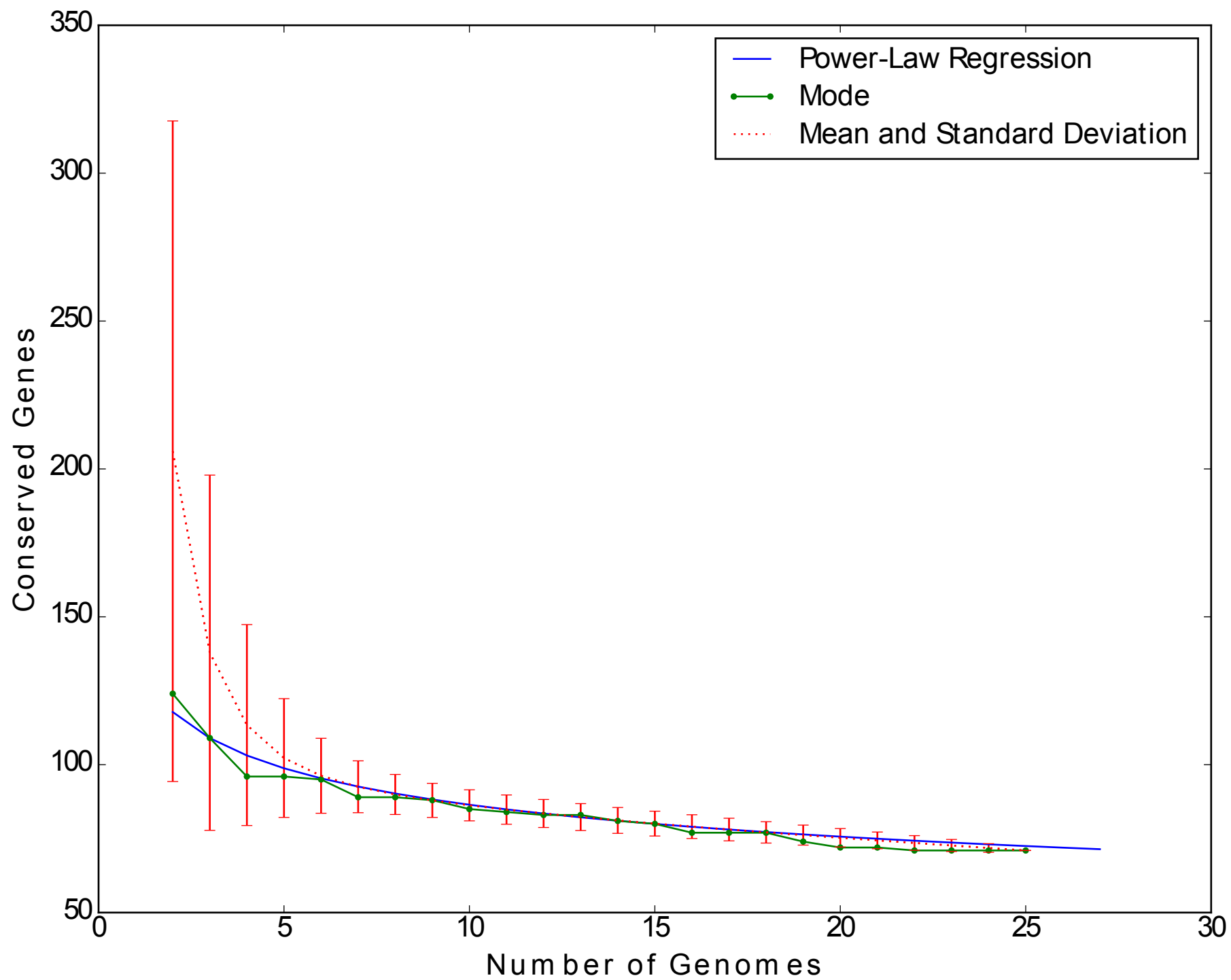

Supplement: FIG S2 [file mbo002173270sf2.pdf]

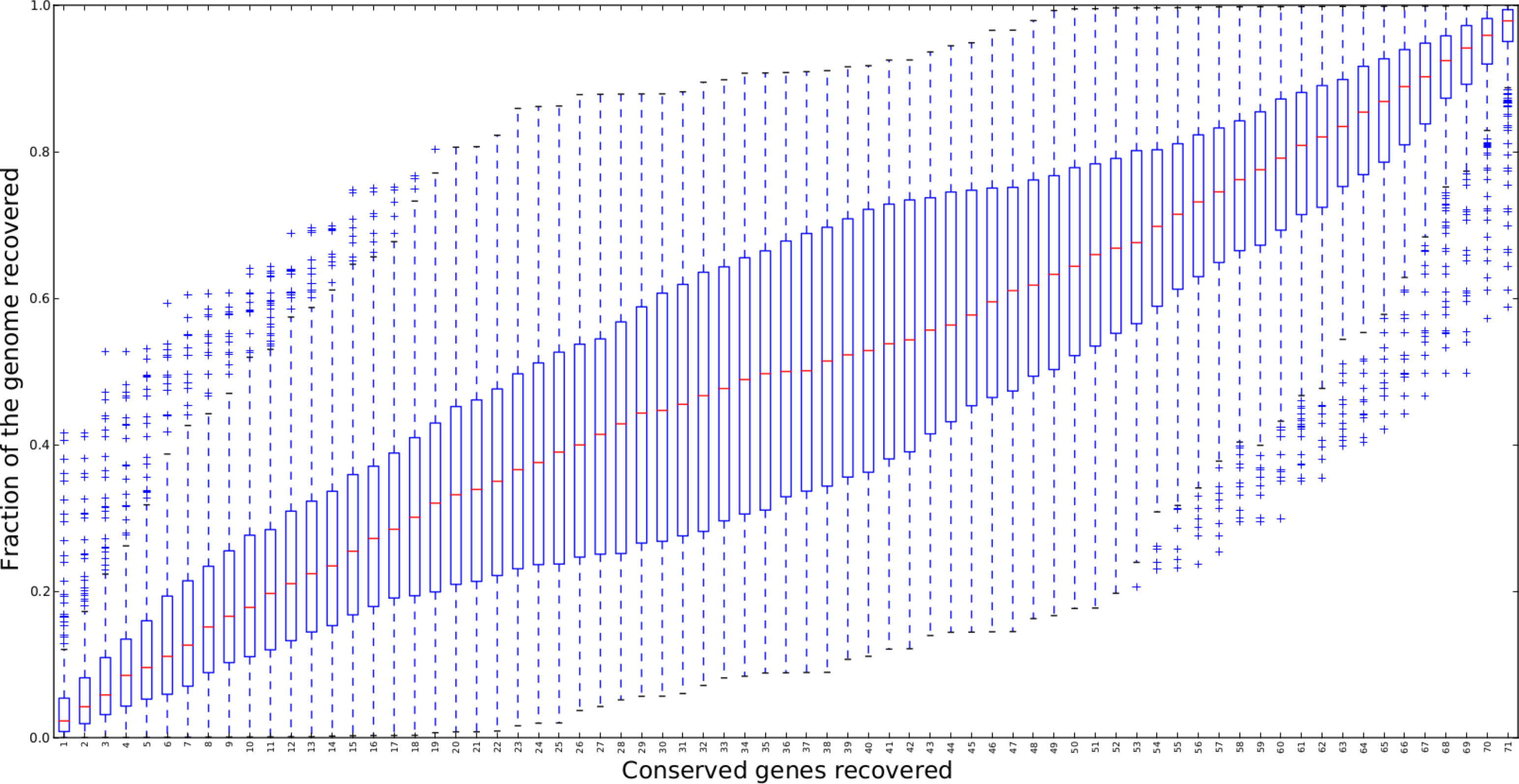

Supplement: FIG S3 [file mbo002173270sf3.pdf]

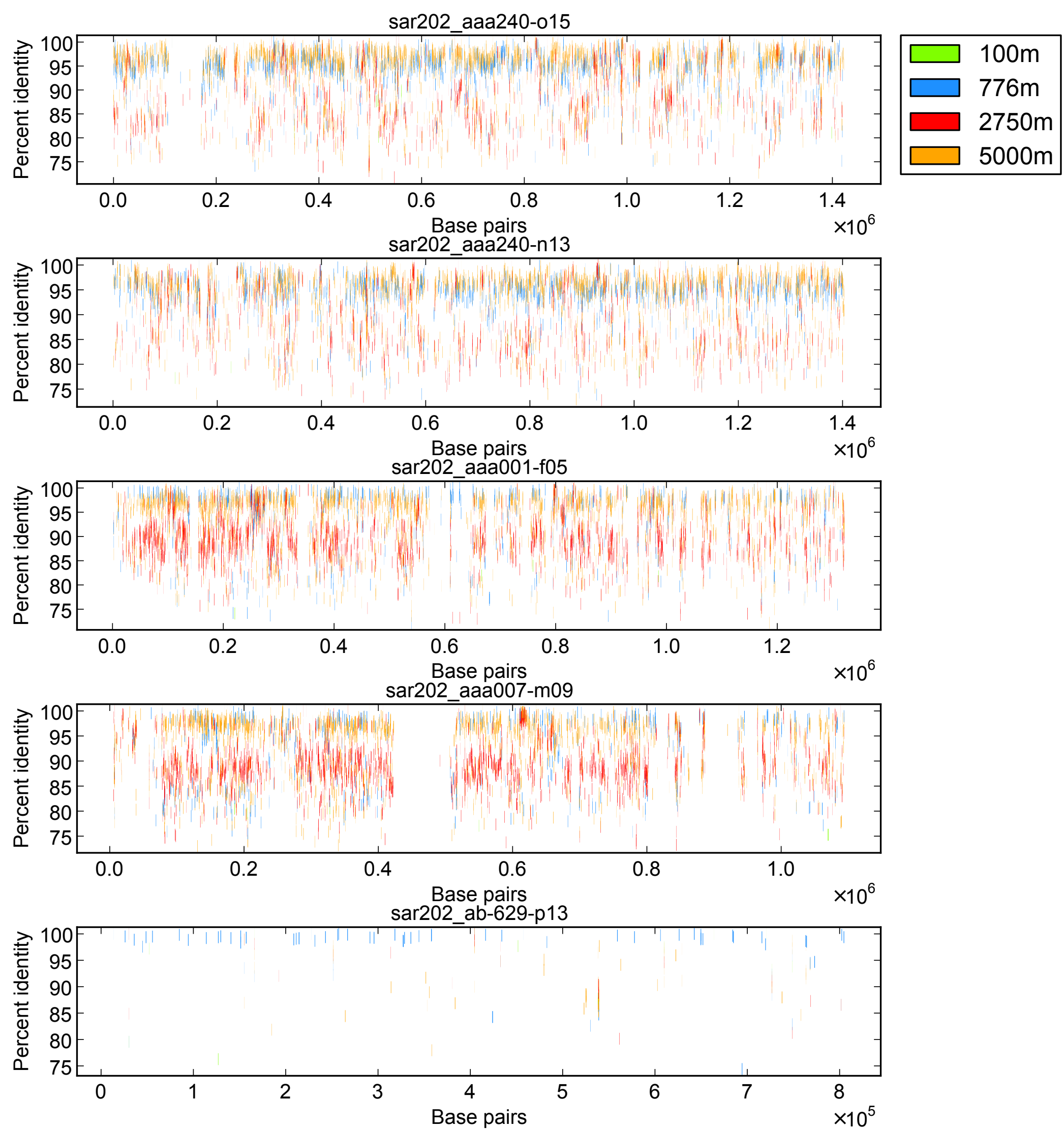

Supplement: FIG S4 [file mbo002173270sf4.pdf]

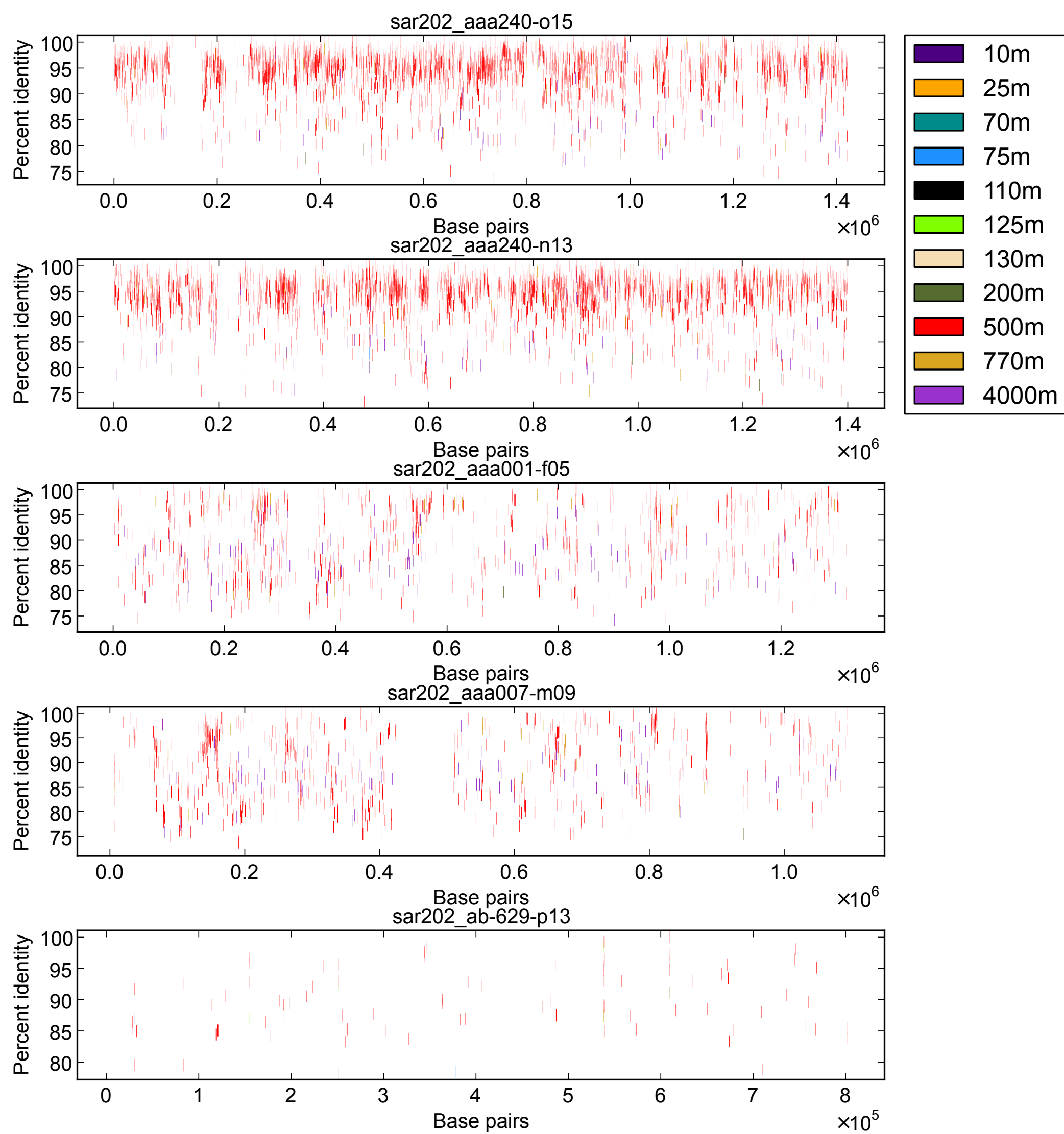

Supplement: FIG S5 [file mbo002173270sf5.pdf]

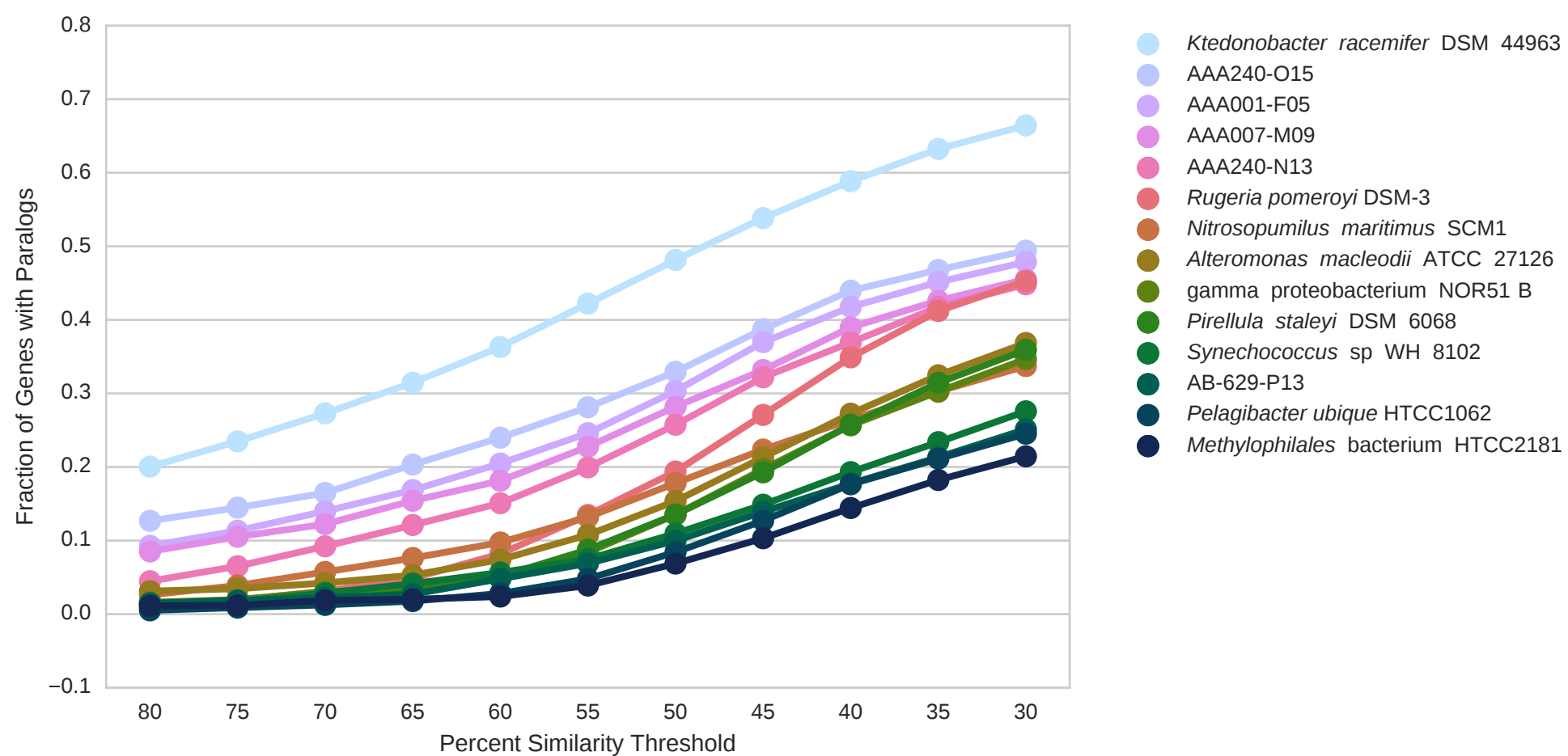

Supplement: FIG S6 [file mbo002173270sf6.pdf]
