# Supplementary material for: SAR202 Genomes from the Dark Ocean Predict Pathways for the Oxidation of Recalcitrant Dissolved Organic Matter
Source: mBio. 2017 Apr 18;8(2):e00413-17. doi: 10.1128/mBio.00413-17 (PMC5395668; doi:10.1128/mBio.00413-17)
Supplement: TABLE S1 [file mbo002173270st1.pdf]

Table S1.) Assembly statistics for 5 SAR202 single-amplified genomes (SAGs). Crossover point (Cp) represent the number of hours into the MDA reaction where the product reached half of its maximum dsDNA fluorescence. Only contigs larger than 2000 bp were used in our analyses.

Assembly Name	Group	No. of Contigs	Total Base Pairs	N50 of Contigs Over 2000 bp	Number of Contigs Larger than N50(2000)	% GC	Cp value	Potential Contamination (CheckM)	Estimated Percent Complete (CheckM)	IMG Accession Number
AAA240-O15	3	93	1,423,799	30,063	13/93	55	13.3	0.0	27.72	2521172610
AAA240-N13	3	181	1,403,147	13,072	28/181	55	13.7	0.0	24.82	2263328036
AAA001-F05	3	133	1,320,825	14,363	24/133	55	14.1	0.0	14.91	2521172608
AAA007-M09	3	70	1,096,525	65,612	5/70	55	0.0	0.2	4.17	2521172609
AB-629-P13	5	39	807,656	37,321	7/39	41	6.2	2.5	2.48	2639762710
