# Supplementary material for: SAR202 Genomes from the Dark Ocean Predict Pathways for the Oxidation of Recalcitrant Dissolved Organic Matter
Source: mBio. 2017 Apr 18;8(2):e00413-17. doi: 10.1128/mBio.00413-17 (PMC5395668; doi:10.1128/mBio.00413-17)
Supplement: TABLE S2 [file mbo002173270st2.pdf]

Table S2.) Reciprocal best blast recruitment of SAR202 single amplified genomes against reads from two metagenomic depth profiles. Note the highest levels of recruitment are from the deepest samples, indicating that these cells likely interact with chemical species found in most remote depths of the ocean.

			**Recruitment (Total Reads Recruited / Percent of Reads Recruited / Average Percent Identity)**																	
**Cell Origin:**			**Hawaii Ocean Time Series**						**South Atlantic Gyre**						**North Atlantic/OMZ**			**-**		
**Site**	**Depth (m)**	**Total Reads**	**AAA240-O15**			**AAA240-N13**			**AAA001-F05**			**AAA007-M09**			**AB-629-P13**			**All**		
MOCA	100	404,351	14	0.00%	88%	9	0.00%	87%	6	0.00%	82%	8	0.00%	80%	14	0.00%	94%	51	0.01%	86%
	776	555,373	1,202	0.22%	94%	1,109	0.20%	94%	626	0.11%	93%	476	0.09%	92%	77	0.01%	97%	3,490	0.63%	94%
	2750	579,381	1,381	0.24%	89%	1,304	0.23%	89%	2,263	0.39%	90%	1,747	0.30%	89%	33	0.01%	94%	6,728	1.16%	90%
	5000	495,906	2,862	0.58%	93%	2,616	0.53%	93%	2,461	0.50%	93%	1,872	0.38%	93%	72	0.01%	92%	9,883	1.99%	93%
**Total**	-	2,035,011	5,459	0.26%	91%	5,038	0.24%	91%	5,356	0.25%	90%	4,103	0.19%	89%	196	0.01%	94%	20,152	0.95%	91%
HOT	10	7,842	4	0.05%	86%	3	0.04%	82%	6	0.08%	83%	1	0.01%	78%	0	0.00%	-	14	0.18%	82%
	25	1,150,263	0	0.00%	-	0	0.00%	-	0	0.00%	-	0	0.00%	-	4	0.00%	91%	4	0.00%	91%
	70	10,999	0	0.00%	-	0	0.00%	-	0	0.00%	-	0	0.00%	-	0	0.00%	-	0	0.00%	-
	75	1,237,622	1	0.00%	83%	5	0.00%	91%	0	0.00%	-	0	0.00%	-	5	0.00%	87%	11	0.00%	87%
	110	473,166	0	0.00%	-	0	0.00%	-	0	0.00%	-	0	0.00%	-	6	0.00%	88%	6	0.00%	88%
	125	501,198	2	0.00%	87%	0	0.00%	-	0	0.00%	-	0	0.00%	-	10	0.00%	92%	12	0.00%	89%
	130	6,812	0	0.00%	-	0	0.00%	-	0	0.00%	-	0	0.00%	-	1	0.01%	94%	1	0.01%	94%
	200	8,286	11	0.13%	84%	9	0.11%	84%	10	0.12%	83%	4	0.05%	81%	0	0.00%	-	34	0.41%	83%
	500	1,537,785	4,654	0.30%	94%	4,450	0.29%	94%	1,349	0.09%	91%	1,059	0.07%	90%	127	0.01%	91%	11,639	0.76%	92%
	770	11,479	37	0.32%	92%	51	0.44%	91%	33	0.29%	92%	23	0.20%	90%	1	0.01%	87%	145	1.26%	90%
	4000	11,229	96	0.85%	86%	94	0.84%	87%	132	1.18%	87%	104	0.93%	88%	1	0.01%	100%	427	3.80%	90%
**Total**	-	4,956,681	4,805	0.15%	87%	4,612	0.16%	88%	1,530	0.16%	87%	1,191	0.11%	85%	115	0.00%	91%	12,293	0.58%	86%
