# Supplementary material for: SAR202 Genomes from the Dark Ocean Predict Pathways for the Oxidation of Recalcitrant Dissolved Organic Matter
Source: mBio. 2017 Apr 18;8(2):e00413-17. doi: 10.1128/mBio.00413-17 (PMC5395668; doi:10.1128/mBio.00413-17)
Supplement: TABLE S3 [file mbo002173270st3.pdf]

Table S3.) Candidate protein sequences found within the SAR202 genomes that potentially code for enzymes or enzyme subunits required by the 3-hydroxypropionate cycle.

Enzyme	Candidate Genes
Acetyl-CoA carboxylase	AAA001-F05: 2521610627, 252161026;<br>AAA007-M09: 2521610950, 2521610951, 2521610952;<br>AAA240-N13: 2264260413, 2264260414, 2521610626, 2264260258, 22664260259;<br>AAA240-O15: 2521612691, 2521612692;<br>AB-629-P13: 2521613197, 2264872648;
Malonyl-CoA reductase	
Propionyl-CoA synthase	AAA001-F05: 2521609775, 2521609932, 2521610279, 2521609568;<br>AAA007-M09: 2521611297, 2521610732;<br>AAA240-N13: 2264260782, 2264261023, 2264260846, 2264260825, 2264260914;<br>AAA240-O15: 2521613155;<br>AB-629-P13: 2264873038
Propionyl-CoA carboxylase	AAA001-F05: 2521610627, 252161026;<br>AAA007-M09: 2521610950, 2521610951, 2521610952;<br>AAA240-N13: 2264260413, 2264260414, 2521610626, 2264260258, 22664260259;<br>AAA240-O15: 2521612691, 2521612692;<br>AB-629-P13: 2521613197, 2264872648;
Methylmalonyl-CoA epimerase	AAA001-F05: 2521610355;<br>AAA240-N13: 2264260289,<br>AAA240-O15: 2521612297,2521612379
Methymalonyl-CoA mutase	AAA240-N13: 2264260840, 226426935;<br>AAA240-O15: 2521612298, 2521612295
Succinyl-CoA:malate-CoA transferase	AAA001-F05: 2521610523;<br>AAA007-M09: 2521610725, 252160772;<br>AAA240-N13: 2264260060;<br>AAA240-O15: 2521612823, 2521612284;<br>AB-629-P13: 2264872365, 2264872881
Succinate dehydrogenase	AAA001-F05: 2521610375
Fumarate hydratase	AAA240-O15: 2521612857;<br>AB-629-P13: 2264873103;
Malyl-CoA/methylmalyl-CoA/citramalyl-CoA trifunctional lyase	AAA007-M09: 2521610711, 2521610713, 2521610712
Methylmalyl-CoA dehydratase	
Mesaconyl-CoA CoA transferase	
Mesaconyl-CoA hydratase	AAA001-F05: 2521609939, 2521609938;<br>AAA240-N13: 2264260672
