# Supplementary material for: SAR202 Genomes from the Dark Ocean Predict Pathways for the Oxidation of Recalcitrant Dissolved Organic Matter
Source: mBio. 2017 Apr 18;8(2):e00413-17. doi: 10.1128/mBio.00413-17 (PMC5395668; doi:10.1128/mBio.00413-17)
Supplement: TEXT S1 [file mbo002173270s1.doc]

**Supplementary Material**

for *SAR202 genomes from the dark ocean predict pathways for the oxidation of recalcitrant dissolved organic matter*

**SUPPLEMENTARY METHODS**

**Single cell sample collection and construction of SAG libraries.** SAGs, ’Chloroflexi bacterium SCGC AAA240-N13’ and ’Chloroflexi bacterium SCGC AAA240-O15’, were isolated from 770 m at the Hawaii Ocean Time-Series (HOTS) sampling site (22° 45'N, 158° 00'W). ’Chloroflexi bacterium SCGC AAA001-F05’ and 'Chloroflexi bacterium SCGC AAA007-M09', were isolated from a depth of 800 m in the South Atlantic Gyre (12°29'S 4°59'W). ’Chloroflexi bacterium SCGC AB-629-P13’, was isolated from the North Atlantic (18°10’N, 41°02’W) at a depth of 511 m Water samples for single cell analyses were collected using Niskin bottles and replicate 1 mL aliquots of water were cryopreserved with 6% glycine betaine (Sigma) and stored at –80ºC (1). Prior to cell sorting, samples with prokaryote cell abundances above 5x10^5 mL-1 were diluted 10x with filter-sterilized field samples and pre-screened through a 70 µm mesh-size cell strainer (BD). For heterotrophic prokaryote detection, diluted subsamples (1-3 mL) were incubated for 10-120 min with SYTO-9 DNA stain (5 µM; Invitrogen). Cell sorting was performed with a MoFlo™ (Beckman Coulter) flow cytometer using a 488 nm argon laser for excitation, a 70 µm nozzle orifice and a CyClone™ robotic arm for droplet deposition into microplates. The cytometer was triggered on side scatter. The “single 1 drop” mode was used for maximal sort purity. Prokaryote cells were separated from eukaryotes, viruses, and detritus based on SYTO-9 fluorescence (proxy to nucleic acid content) and light side scatter (proxy to particle size) (2). *Synechococcus* cells were excluded, based on their autofluorescence signal. Target cells were deposited into 384-well plates containing 600 nL per well of either 1x TE buffer or prepGEM™ Bacteria (Zygem) reaction mix and stored at –80ºC until further processing. Of the 384 wells, 315 were dedicated for single cells, 66 were used as negative controls (no droplet deposited) and 3 received 10 cells each (positive controls). The accuracy of droplet deposition was determined by depositing 10 µm fluorescent beads into 384-well plates and the results checked by microscopically verifying the presence of beads in the plate wells. Of the 2-3 plates examined each sort day, with one bead deposited per well, fewer than 2% of wells were found to contain no bead and 0.4% to contain more than one bead. The latter is most likely caused by co-deposition of two beads attached to each other, which at certain orientation may have similar optical properties to single beads.

Prior to cell sorting, the instrument and the workspace were decontaminated for DNA as previously described (3). DNA contaminants in all MDA reagents were cross-linked by a UV treatment in Stratalinker (Stratagene) (4). During UV treatment, reagents were placed on ice to avoid overheating. An empirical optimization of the UV exposure was performed to remove all detectable contaminants without inactivating the reaction. Cell sorting and MDA setup were performed in a HEPA-filtered environment. As a quality control, the kinetics of all MDA reactions were monitored by measuring the SYTO-9 fluorescence using either LightCycler 480 (Roche) or FLUOstar Omega (BMG). The critical point (Cp) was determined for each MDA reaction as the time required to produce half of the maximal fluorescence. The Cp is inversely correlated to the amount of DNA template (5).

Sorted cells were lysed and their DNA was denatured using cold KOH (6). Genomic DNA from the lysed cells was amplified using multiple displacement amplification (MDA) (6, 7) in 10 µL final volume. The MDA reactions contained 2 U/µL Repliphi polymerase (Epicentre), 1x reaction buffer (Epicentre), 0.4 mM each dNTP (Epicentre), 2 mM DTT (Epicentre), 500 µM phosphorthioated random hexamers (IDT) and 1 µM SYTO-9 (Invitrogen) (all final concentration). The MDA reactions were run at 30°C for 12-16 h, and then inactivated by a 15 min incubation at 65°C. Amplified genomic DNA was stored at -80°C until further processing. We refer to the MDA products originating from individual cells as single amplified genomes (SAGs). (3–5)

**PCR screening of SAG libraries.** MDA products were diluted 50-fold in TE buffer and 500 nL aliquots of diluted MDA product served as template DNA in 5 µL final volume real-time PCR screens targeting bacterial small subunit rRNA genes using primers 27F´ and 907R (8, 9). All PCR reactions were performed using LightCycler 480 SYBR Green I Master Mix (Roche) and the Roche LightCycler® 480 II real-time thermal cycler. Forward (5´–GTAAAACGACGGCCAGT–3´) and reverse (5´–CAGGAAACAGCTATGACC–3´) M13 sequencing primers were added to the 5´ ends of each target primer pair to aid direct sequencing of PCR products. All PCR reactions were run for 40 cycles at the appropriate annealing temperature, followed by melting curve analysis performed as follows: 95°C for 5 s, 52°C for 1 min, and a continuous temperature ramp (0.11°C/s) from 52 to 97°C. Real-time PCR kinetics and amplicon melting curves served as proxies for detecting SAGs positive for 16S rRNA genes. New, 20 µL PCR reactions were set up for all PCR-positive SAGs and amplicons were sequenced from both ends using Sanger technology by Beckman Coulter Genomics. Single cell sorting, whole genome amplification, real-time PCR screens and PCR product sequence analyses were performed at the Bigelow Laboratory Single Cell Genomics Center (http://scgc.bigelow.org).

**SAG sequencing and assembly** A total of 5 SAGs belonging to the SAR202 Chloroflexi cluster were chosen for whole genome sequencing and assembly based on multiple displacement amplification (MDA) kinetics, phylogenetic affiliation of small subunit rRNA genes, as well as geographic location and water depth. Two different approaches were employed for whole genome sequencing (WGS): 1: A combination of Illumina and 454 shotgun sequencing ('Chloroflexi bacterium SCGC AAA240-N13') as described in Swan et al., 2011 (10); and 2: Illumina-only sequencing ('Chloroflexi bacterium SCGC AB-629-P13', 'Chloroflexi bacterium SCGC AAA001-F05', 'Chloroflexi bacterium SCGC AAA007-M09' and 'Chloroflexi bacterium SCGC AAA240-O15') as described in Swan et al., 2013 and Wilkins et al., 2014 (11, 12). In addition, three different approaches were employed for genome assembly and each is described below.

**'Chloroflexi bacterium SCGC AAA240-N13' assembly.** A combination of Illumina and 454 shotgun sequencing was performed on the single cell re-MDA products of SAG AAA240-N13 at the DOE Joint genome Institute (JGI). For Illumina sequencing, normalized 0.3 Kbp shotgun libraries were constructed. Briefly, 3 µg MDA product was sheared in 100 µl using the Covaris E210 with the setting of 10% duty cycle, intensity 5, and 200 cycle per burst for 3 min per sample, and the fragmented DNA was purified using QIAquick columns (Qiagen) according to the manufacturer's instructions. The sheared DNA was end-repaired and A-tailed according to the Illumina standard PE protocol, and purified using the MinElute PCR Purification Kit (Qiagen) with a final elution in 12 µl of Buffer EB. After quantification using a Bioanalyzer DNA 1000 chip (Agilent), the fragments were ligated to Illumina adaptors according to the Illumina standard PE protocol, followed by a purification step of the ligation product using AMPure SPRI beads. The Illumina libraries were quantified using a Bioanalyzer DNA High Sensitivity chip (Agilent) and 300 ng of DNA (in 6 µl) then underwent normalization using the Duplex-Specific Nuclease (DSN) Kit (Axxora) (13). For normalization, the dsDNA was denatured for 3 min at 98°C, following a hybridization step at 68°C for 5h and DSN treatment at 68°C for 20 min. Normalized libraries were amplified by PCR for 12 cycles, gel-purified and QC assessed on a Bioanalyzer DNA High Sensitivity chip (Agilent), and then sequenced using an Illumina GAIIx sequencer. For 454 shotgun sequencing, a 4Kbp paired-end library was constructed and sequenced. All general aspects of and detailed protocols for library construction and sequencing can be found at the JGI website (http://www.jgi.doe.gov/).

The following steps were performed for genome assembly: 1) normalized Illumina reads were assembled using Velvet version 1.1.02 (14). The VelvetOptimiser script (version 2.1.7) was used with default optimization functions (N50 for k-mer choice, total number of base pairs in large contigs for cov_cutoff optimization). 2) Next, Illumina contigs larger than 800 bp were shredded into 1 Kbp pieces with 200 bp overlaps. 3) Illumina shreds and raw 454 pyrosequence reads were then assembled using the 454 Newbler assembler version 2.4 (Roche). The 454 contigs were subject to another round of assembly using Sequencher software version 4.10.1 (Gene Codes). Ambiguities were trimmed off the ends and contigs overlapping by at least 100 bp and 98% sequence identity were merged into larger contigs.

**'Chloroflexi bacterium SCGC AB-629-P13' WGS and assembly.** A draft genome of 'Chloroflexi bacterium SCGC AB-629-P13' was generated at the DOE Joint genome Institute (JGI) using Illumina technology. An Illumina standard shotgun library was constructed and sequenced using the Illumina HiSeq 2000 platform. All general aspects of library construction and sequencing performed at the JGI can be found at http://www.jgi.doe.gov. All raw Illumina sequence data was passed through DUK, a filtering program developed at JGI, which removes known Illumina sequencing and library preparation artifacts.

The following steps were then performed for assembly: 1) filtered Illumina reads were assembled using Velvet v. 1.1.04 (14), 2) 1–3 kbp simulated paired end reads were created from Velvet contigs using wgsim (http://github.com/lh3/wgsim), 3) Illumina reads were assembled with simulated read pairs using Allpaths–LG v. r41043 (15). Parameters for assembly steps were: 1) Velvet: 63 -shortPaired and velvetg: -very clean yes -export -Filtered yes -min contig lgth 500 -scaffolding no -cov cutoff 10, 2) wgsim: -e 0 -1 100 -2 100 -r 0 -R 0 -X 0, 3) Allpaths: -LG PrepareAllpathsInputs: PHRED 64=1 PLOIDY=1 FRAG COVERAGE=125 JUMP COVERAGE=25 LONG JUMP COV=50, RunAllpathsLG: THREADS=8 RUN=std shredpairs TARGETS=standard VAPI WARN ONLY=True OVERWRITE=True.

**Chloroflexi bacterium AAA001-F05, AAA007-M09 and AAA240-O15 assemblies.** Genomic library preparation and sequencing of SAGs AAA001-F05, AAA007-M09 and AAA240-O15 was performed at the Oregon State University’s Center for Genome Research (cgrb.oregonstate.edu). SAG genomic DNA was sheared using an S220 focused ultrasonicator (Covaris) and gel fractionated for 450 bp fragments. Illumina sequencing libraries were prepared using TruSeq reagents and protocols (Illumina). Then, 2 x 100 bp paired-end reads were sequenced using the Illumina HiSeq 2000 platform (Illumina). For each sample, sequence data were quality trimmed (Q,0) and reads with greater than 5% ambiguities were removed using the NGS Toolkit (16). The sequence data were then digitally normalized to reduce over-represented and under-represented sequences (17). Normalized reads were assembled by the SCGC using SPAdes v.2.2.1 (18) using the following settings: data flagged as single-cell, error correction max iterations 2 and iterative k-mer sizes 21,33,55. Assembled contigs shorter than 2000 bp were removed.

Whole-genome sequence data for all SAR202 cluster SAGs are available in IMG under accession numbers listed in Supplementary Table 1.

**SAG whole genome sequence quality control.** Each raw sequence data set was screened against all finished bacterial and archaeal genome sequences (downloaded from NCBI) and the human genome to identify potential contamination in the sample. Reads were mapped against reference genomes with bwa version 0.5.9 (19) using default parameters (96% identity threshold). Additionally, CheckM (20) was used with amino acid coding sequences to assess contamination and assembly completion. None of the libraries showed significant contamination. Additionally, gene sequences of the final assemblies (see below) were compared against the GenBank nr database by BLASTX and taxonomically classified using MEGAN (21). To further verify the absence of contaminating sequences in the assemblies, tetramer frequencies were extracted from the assembled contigs and Principal Component Analysis (PCA) of the assembled contigs was conducted in order to identify outliers (10, 22). Contigs representing extremes on the first eight PCs were examined for their closest blastx hits against the National Center for Biotechnology Information (NCBI) nr database as well as other assembled genomes sequenced at the same time in order to identify possible contaminants.

**SAG annotation.** Initial annotation was performed by the US Department of Energy’s Joint Genome Institute (JGI). The gene modeling program Prodigal (http://prodigal.ornl.gov/) was run on the draft single cell genomes, using default settings that permit overlapping genes and using ATG, GTG, and TTG as potential starts. The resulting protein translations were compared to the GenBank non-redundant database (NR), the Swiss-Prot/TrEMBL, Pfam, TIGRFam, Interpro, KEGG, and COGs databases using BLASTP or HMMER. From these results, product assignments were made. Initial automated annotation was performed by the Joint Genome Institute’s Integrated Microbial Genomes Resource and initial criteria for automated functional assignment set priority based on TIGRFam, Pfam, COG, Interpro profiles, pairwise BLAST versus Swiss-Prot/TrEMBL, and KO groups. The annotation was imported into the Joint Genome Institute Integrated Microbial Genomes (IMG; <http://img.jgi.doe.gov/cgi-bin/pub/main.cgi>) (23, 24).

Following annnotation by the JGI, ll predicted amino acid sequences were additionally annotated using the Sifting Families resource (Sfams) (25) of protein families. Sfams is a computationally derived global database of microbial protein families, and contains corresponding full-length amino acid alignments and Markov models for each family. Hmmscan (26) was used to compare all Sfam families to all SAR202 amino acid sequences (options: --incE 0.001) The results of this search were parsed using python (27) and each amino acid sequence was assigned to the best hitting Sfam model with at least 80% reciprocal alignment by length.

**Phylogenomic Inference.** Amino acid sequences from 25 Chloroflexi genomes were collected from the Integrated Microbial Genomes (IMG) database for a phylogenomic analysis. This analysis includes the following finished or permanent draft genomes: *Anaerolinea thermophila* UNI-1, *Caldilinea aerophila* STL-6-O1 DSM 14535, *Candidatus Chlorothrix halophila, Chloroflexus aggregans DSM* 9485*, Chloroflexus aurantiacus* J-10-fl, *Chloroflexus sp.* T88, *Chloroflexus sp* Y-396-1, *Chloroflexus sp.* Y-400-fl, *Dehalococcoides ethenogenes* 195, *Dehalococcoides mccartyi* BAV1*, Dehalococcoides mccartyi* BTF08, *Dehalococcoides mccartyi* CBDB1, *Dehalococcoides mccartyi* DCMB5, *Dehalococcoides mccartyi* GT, *Dehalococcoides mccartyi* GY50, *Dehalococcoides mccartyi* VS, *Dehalogenimonas lykanthroporepellens* BL-DC-9, *Herpetosiphon aurantiacus* DSM 785, *Ktedonobacter racemifer SOSP1-21 DSM* 44963, *Nitrolancetus hollandicus* Lb, *Oscillochloris trichoides* DG6, *Roseiflexus castenholzii* HLO8 DSM 13941, *Roseiflexus sp.* RS-1, *Sphaerobacter thermophilus* 4ac11 DSM 20745, and *Thermomicrobium roseum* DSM 5159. Additionally, the analysis included the following SAGs and metagenomic assemblies from more recent studies: Dsc1 and DscP2 (28), RBG1351 (29), and DEH-J10 (30). All 5 of the SAR202 SAGs from this study were also included: 'Chloroflexi bacterium SCGC AAA001-F05', 'Chloroflexi bacterium SCGC AAA007-M09', 'Chloroflexi bacterium SCGC AAA240-N13', 'Chloroflexi bacterium SCGC AAA240-O15', and 'Chloroflexi bacterium SCGC AB-629-P13' SAGs. *Pirellula staleyi DSM 6068* was included as an outgroup. Phylogenomic trees and orthology were determined using a re-implementatiion of the Hal pipeline workflow (31–33), in which legacy BLAST (34) was replaced with BLAST+ 2.2.26 (35), mcl was updated to version 12-068 (36), muscle to version 3.8.31 (37, 38), ProtTest to version 3.4 (39), RAxML version 8.0.22 (40), and ZORRO (41) was substituted for GBLOCKS (42, 43) for probabilistic masking. Homology was detected with an all vs all blastp using the parameters described in Moreno-Hagelsieb and Latimer, 2007 (44) to maximize detection of orthologs. Results were limited to the number of genomes included in the analysis as described in Robbertse et al., 2011 (32). Hits were subjected to clustering using the Markov clustering algorithm (mcl) (36) using the same edge-weighting scheme and range of inflation parameters (1.1, 1.2, 1.3, 1.4, 1.5, 1.7, 2.0, 2.5, 3.0, 3.5, 4.0, 4.5 and 5.0.) as in previous studies (32) Clusters of orthologs were selected using the criterion outlined in Robbertse et al., 2011 (32). beginning with the most conservative (highest) inflation parameter and moving to the most inclusive (lowest) inflation parameter. Clusters were selected that contained a single gene from each included organism and where the best hit for each gene was included in that cluster. Clusters were selected iteratively to maximize the number of organisms included in each gene cluster, and only clusters exceeding a specified acceptable missing data value were used in the final phylogenomic analysis. Here, clusters that contained representative genes from at least 80 % of the members were included in the final alignment. Clusters with fully identical sequences were also removed, as they provided no phylogenetic information. Amino acid sequences of selected gene clusters were aligned using the MUSCLE (37, 38) aligner. Alignments were masked using the probabilistic masker ZORRO (41), masking columns with weight less than 0.5, as per the suggestion of the authors. ProtTest 3 (39) was used to establish the best model for phylogenetic inference, testing the LG, WAG, VT, JTT, Blosum62, CpREV, RtREV, DCMut, Dayhoff, FLU, HIVb, MtREV, HIVw, MtArt, and MtMam amino acid substitution models. The best model was assessed using the AIC, BIC and AICc assement critera included in ProtTest. When parsing the ProtTest results, if a consensus for the top-scoring model was reached between two or more of the assessment criteria, that model was selected. In the event of different models being chosen by each assessment criteria,the model with the best confidence score under any of the three assessment criteria was selected. In the above case, if there was a tie between confidence scores, the model was selected by the BIC, AIC, and then AICc criterion, in that order. Following model selection, selected and masked alignments were concatenated, with missing data replaced with null characters. The selected models were applied to the appropriate partitions of this super alignment. Using RAxML, a maximum likelihood tree was computed using the masked super alignment and model partitions, using the rapid hill climbing method (options: -f d) and 10 starting trees (options: -N 10). 1000 bootstraps were calculated in 42 batches of less than or equal to 24 using the rapid bootstrapping option (-x) and were assigned to the previously calculated maximum-likelihood tree (options: -f b). Throughout the pipeline process, a series of in-house Python wrapper scripts made use of the Biopython library (27) to organize job-queues and between-job filtering of results, including preparing sequences and intermediate files for job submission, filtering of Markov clusters, masking of alignments, parsing of ProtTest results, and super-alignment concatenation.

**Estimated genome size.** Genome size estimates were obtained using a set of core genes derived through a combinatorial core genome analysis of the entire Chloroflexi phylum (22, 45–47). Only permanent draft/fully sequenced organisms were used in this analysis, previously mentioned single-cell sequences and metagenomic assemblies were removed to avoid interference from their inherently incomplete nature. Clusters of genes generated in the first steps of the phylogenomic analysis (prior to filtering) were used as a starting point. Clusters of absolutely conserved single-copy genes were identified for every combination of genomes for all set sizes from 2 to 25 genomes. Following the combinatorial analysis, a power law regression on the of all combinations was performed to predict any change in the conserved single-copy gene set that might result from the addition of subsequent genomes (See Supplementary figure 2).

To account for uneven distributions of single-copy genes within genomes, and to predict a range of genome recovery from conserved gene recovery, an iterative rolling resampling approach similar to that taken in Woyke et al., 2009 (22) was used. Each of the concatenated genomes (represented as a circle) was divided into 20º sections, and was repeatedly scanned for the locations of conserved genes, beginning at 0 and moving forward 20º for each subsequent scan, for a total of 18 scans. The fraction of the genome scanned vs. the number of conserved genes discovered was recorded for each conserved gene, for each starting point (See Supplementary Figure 3). It can be seen in supplementary figure 3 that the predictive abilities of the conserved gene sets become less reliable in the tail regions, effectively increasing the chances of over-estimating completion with lower numbers of recovered conserved genes and under-estimating completion with higher numbers. Presumably, this is due to the aforementioned clustering of conserved gene sets within the genome.

Combinatorial identification of conserved single-copy gene clusters and iterative discovery of conserved single-copy genes were calculated in Python, and additionally, power-law regression was performed using the scipy.optimize curve-fit functionality in the SciPy package for scientific data processing.

**Global phylogeny of FMNOs.** Following re-assessment/re-annotation of all 5 genomes using the Sfams resource, the coding sequences of 48 genes with best hits to Sfam 4832 were extracted. These genes were commonly annotated as either a luciferase-like monooxygenases or as flavin/F420-dependent oxidoreductases.

Reasoning that the highly subdivided sequence space of the Sfam families might lead us to miss families of diverged paralogs, we attempted to identify any additional paralogous genes or gene families within the SAR202 genomes. Initially the HHSearch program for Markov model comparisons (48) was used to recruit similar Markov models from the Sfams database. HHsearch was run against the entire Sfams database of alignments, and Markov models for matches sharing at least 50% matching columns and having an e-value of at least 0.001 were extracted. All genes that had been matched to one of these models in the previous Sfams analysis were identified. To find orthologous gene families, the set of matching Markov models was used with hmmsearch tool of HMMer 3 (options: --incE 0.01) to recruit all similar sequences from the manually curated SwissProt/Uniprot database (49), as well as all similar bacterial proteins from the RefSeq65 database of genome sequences (50). Known single-cell genomes were excluded from this search. To confirm orthology, these recruited sequences were then reciprocally searched against all Sfams families. For each sequence, the top-hitting family with at least 80% alignment across both the sequence and the model was used as the top hit. Sequences whose top hit was within the original set of Sfams were kept for further analysis.

Due to the large number of sequences returned, the full set of sequences was dereplicated using UCLUST program (51) to reduce the original sequence set to a number of consensus sequences representing sequences with 80% alignment coverage and at least 50% identity (options: -cluster_fast, -query_cov 0.80, -target_cov 0.80, -id 0.50, -consout). Following dereplication, this set of consensus sequences was aligned using MUSCLE. Probabilistic masking of the alignment was performed using ZORRO. Columns with a weight of less than 0.5 were removed the alignment using the Biopython AlignIO module; (27), as per recommendation of the authors of the software (41). A final tree was created using FastTree (52); options: -wag -gamma).

**Reciprocal best-blast recruitment.** Genome sequences for all SAR202 SAGs were compared to both the HOTS and MOCA metagenomic depth profiles using a reciprocal best-blast approach. Scaffold sequences from each of the five sequenced SAGs were compared with all the sequences from each of the target depth profiles and all sequences with an e value lower than 1 were kept (-evalue 1). –max_target_seqs was set to an artificially high value (1e9) to ensure all possible hits were returned in the initial search. Following the initial search, all returned sequences were then reciprocally compared to a copy of the Refseq65 database with single-cell genomes removed and with the SAR202 sequences in question added. The highest scoring hit for each sequence was kept (-max_target_seqs 1). All hits that matched scaffolds of the SAR202 SAG in question were kept as reciprocal best hits. This analysis was performed independently for the HOTS and MOCA datasets. Post processing and plotting of the data was performed in python using the matplotlib plotting library (53).

**Assessment of Paralogy.** Amino acid sequences for all SAR202 SAGs, *Ktedonobacter racemifer DSM 44963,* and a number of other representative marine organisms, including *Silicibacter pomeroyi* DSS­3, *Nitrosopumilus maritimus* SCM1, *Alteromonas macleodii* ATCC 27126, gamma proteobacterium NOR51-B, *Pirellula staleyi* DSM 6068, *Synechococcus* sp. WH 8102, *Pelagibacter ubique* HTCC1062, and Methylophilales bacterium HTCC2181 were used to confirm our assessment that the SAR202 genomes show higher-than-normal levels of paralogy with comparison to other marine organims. The amino acid set for each organism was compared to itself using blast (parameters: -evalue 0.1 -seg yes -soft_masking true -use_sw_tback), and paralogy was assessed using an increasingly relaxed similarity threshold. At each similarity threshold, sequences with top-hitting paralogs above the threshold were tallied and normalized to the total number of genes in the genome (Supplementary Figure 7). The group III SAR202 and *Ktedonobacter* genomes showed consistently higher numbers of paralogs at each similarity threshold, compared to the other organisms in the analysis. The SAR202 Group IV (AB-629-P13) appeared to have very low levels of paralogs, more similar to streamlined organisms such as *Pelagibacter ubique*.

**REFERENCES:**

1. **Cleland D, Krader P, McCree C, Tang J, Emerson D.** 2004. Glycine betaine as a cryoprotectant for prokaryotes. J Microbiol Methods 58:31–38.

2. **Giorgio PA del, Bird DF, Prairie YT, Planas D.** 1996. Flow Cytometric Determination of Bacterial Abundance in Lake Plankton with the Green Nucleic Acid Stain SYTO 13. Limnol Oceanogr 41:783–789.

3. **Stepanauskas R, Sieracki ME.** 2007. Matching phylogeny and metabolism in the uncultured marine bacteria, one cell at a time. Proc Natl Acad Sci 104:9052–9057.

4. **Woyke T, Sczyrba A, Lee J, Rinke C, Tighe D, Clingenpeel S, Malmstrom R, Stepanauskas R, Cheng J-F.** 2011. Decontamination of MDA Reagents for Single Cell Whole Genome Amplification. PLoS ONE 6:e26161.

5. **Zhang T, Fang HHP.** 2006. Applications of real-time polymerase chain reaction for quantification of microorganisms in environmental samples. Appl Microbiol Biotechnol 70:281–289.

6. **Raghunathan A, Ferguson HR, Bornarth CJ, Song W, Driscoll M, Lasken RS.** 2005. Genomic DNA Amplification from a Single Bacterium. Appl Environ Microbiol 71:3342–3347.

7. **Dean FB.** 2002. Comprehensive human genome amplification using multiple displacement amplification. Proc Natl Acad Sci 99:5261–5266.

8. **Casamayor EO, Schäfer H, Bañeras L, Pedrós-Alió C, Muyzer G.** 2000. Identification of and spatio-temporal differences between microbial assemblages from two neighboring sulfurous lakes: comparison by microscopy and denaturing gradient gel electrophoresis. Appl Environ Microbiol 66:499–508.

9. **Lane D.** 1991. 16S/23S rRNA sequencing. Nucleic Acid Tech Bact Syst 125–175.

10. **Swan BK, Martinez-Garcia M, Preston CM, Sczyrba A, Woyke T, Lamy D, Reinthaler T, Poulton NJ, Masland EDP, Gomez ML, Sieracki ME, DeLong EF, Herndl GJ, Stepanauskas R.** 2011. Potential for Chemolithoautotrophy Among Ubiquitous Bacteria Lineages in the Dark Ocean. Science 333:1296–1300.

11. **Swan BK, Tupper B, Sczyrba A, Lauro FM, Martinez-Garcia M, González JM, Luo H, Wright JJ, Landry ZC, Hanson NW.** 2013. Prevalent genome streamlining and latitudinal divergence of planktonic bacteria in the surface ocean. Proc Natl Acad Sci 110:11463–11468.

12. **Wilkins MJ, Kennedy DW, Castelle CJ, Field EK, Stepanauskas R, Fredrickson JK, Konopka AE.** 2014. Single-cell genomics reveals metabolic strategies for microbial growth and survival in an oligotrophic aquifer. Microbiology 160:362–372.

13.  **Bogdanova EA, Shagina IA, Mudrik E, Ivanov I, Amon P, Vagner LL, Lukyanov SA, Shagin DA.** 2009. DSN Depletion is a Simple Method to Remove Selected Transcripts from cDNA Populations. Mol Biotechnol 41:247–253.

14. **Zerbino DR, Birney E.** 2008. Velvet: Algorithms for de novo short read assembly using de Bruijn graphs. Genome Res 18:821–829.

15. **Gnerre S, MacCallum I, Przybylski D, Ribeiro FJ, Burton JN, Walker BJ, Sharpe T, Hall G, Shea TP, Sykes S, Berlin AM, Aird D, Costello M, Daza R, Williams L, Nicol R, Gnirke A, Nusbaum C, Lander ES, Jaffe DB.** 2011. High-quality draft assemblies of mammalian genomes from massively parallel sequence data. Proc Natl Acad Sci 108:1513–1518.

16. **Patel RK, Jain M.** 2012. NGS QC Toolkit: a toolkit for quality control of next generation sequencing data. PloS One 7:e30619.

17. **Brown TC, Camille S, Sheneman L, Rosenthal J, Howe A.** 2013. khmer-protocols documentation. figshare.

18.  **Bankevich A, Nurk S, Antipov D, Gurevich AA, Dvorkin M, Kulikov AS, Lesin VM, Nikolenko SI, Pham S, Prjibelski AD, Pyshkin AV, Sirotkin AV, Vyahhi N, Tesler G, Alekseyev MA, Pevzner PA.** 2012. SPAdes: A New Genome Assembly Algorithm and Its Applications to Single-Cell Sequencing. J Comput Biol 19:455–477.

19.  **Li H, Durbin R**. 2009. Fast and accurate short read alignment with Burrows-Wheeler transform. Bioinformatics 25:1754–1760.

20. P**arks DH, Imelfort M, Skennerton CT, Hugenholtz P, Tyson GW.** 2015. CheckM: assessing the quality of microbial genomes recovered from isolates, single cells, and metagenomes. Genome Res 25:1043–1055.

21. Huson DH, Auch AF, Qi J, Schuster SC. 2007. MEGAN analysis of metagenomic data. Genome Res 17:377–386.

22. **Woyke T, Xie G, Copeland A, González JM, Han C, Kiss H, Saw JH, Senin P, Yang C, Chatterji S, Cheng J-F, Eisen JA, Sieracki ME, Stepanauskas R.** 2009. Assembling the Marine Metagenome, One Cell at a Time. PLoS ONE 4:e5299.

23. **Markowitz VM, Chen I-MA, Palaniappan K, Chu K, Szeto E, Grechkin Y, Ratner A, Jacob B, Huang J, Williams P, Huntemann M, Anderson I, Mavromatis K, Ivanova NN, Kyrpides NC.** 2012. IMG: the integrated microbial genomes database and comparative analysis system. Nucleic Acids Res 40:D115–D122.

24. **Markowitz VM, Korzeniewski F, Palaniappan K, Szeto E, Werner G, Padki A, Zhao X, Dubchak I, Hugenholtz P, Anderson I, Lykidis A, Mavromatis K, Ivanova N, Kyrpides NC.** 2005. The integrated microbial genomes (IMG) system. Nucleic Acids Res 34:D344–D348.

25. **Sharpton T, Jospin G, Wu D, Langille M, Pollard K, Eisen J.** 2012. Sifting through genomes with iterative-sequence clustering produces a large, phylogenetically diverse protein-family resource. BMC Bioinformatics 13:264.

26.  **Eddy SR.** 2011. Accelerated Profile HMM Searches. PLoS Comput Biol 7:e1002195.

27.  **Chapman B, Chang J.** 2000. Biopython: Python tools for computational biology. ACM Sigbio Newsl 20:15–19.

28. **Kaster A-K, Mayer-Blackwell K, Pasarelli B, Spormann AM.** 2014. Single cell genomic study of Dehalococcoidetes species from deep-sea sediments of the Peruvian Margin. ISME J.

29. **Hug LA, Castelle CJ, Wrighton KC, Thomas BC, Sharon I, Frischkorn KR, Williams KH, Tringe SG, Banfield JF. 2013.** Community genomic analyses constrain the distribution of metabolic traits across the Chloroflexi phylum and indicate roles in sediment carbon cycling. Microbiome 1:22.

30. **Wasmund K, Schreiber L, Lloyd KG, Petersen DG, Schramm A, Stepanauskas R, Jørgensen BB, Adrian L.** 2014. Genome sequencing of a single cell of the widely distributed marine subsurface Dehalococcoidia, phylum Chloroflexi. ISME J 8:383–397.

31. **Floudas D, Binder M, Riley R, Barry K, Blanchette RA, Henrissat B, Martinez AT, Otillar R, Spatafora JW, Yadav JS, Aerts A, Benoit I, Boyd A, Carlson A, Copeland A, Coutinho PM, de Vries RP, Ferreira P, Findley K, Foster B, Gaskell J, Glotzer D, Gorecki P, Heitman J, Hesse C, Hori C, Igarashi K, Jurgens JA, Kallen N, Kersten P, Kohler A, Kues U, Kumar TKA, Kuo A, LaButti K, Larrondo LF, Lindquist E, Ling A, Lombard V, Lucas S, Lundell T, Martin R, McLaughlin DJ, Morgenstern I, Morin E, Murat C, Nagy LG, Nolan M, Ohm RA, Patyshakuliyeva A, Rokas A, Ruiz-Duenas FJ, Sabat G, Salamov A, Samejima M, Schmutz J, Slot JC, St. John F, Stenlid J, Sun H, Sun S, Syed K, Tsang A, Wiebenga A, Young D, Pisabarro A, Eastwood DC, Martin F, Cullen D, Grigoriev IV, Hibbett DS**. 2012. The Paleozoic Origin of Enzymatic Lignin Decomposition Reconstructed from 31 Fungal Genomes. Science 336:1715–1719.

32.  **Robbertse B, Yoder RJ, Boyd A, Reeves J, Spatafora JW.** 2011. Hal: an Automated Pipeline for Phylogenetic Analyses of Genomic Data. PLoS Curr 3:RRN1213.

33. **Thrash JC, Temperton B, Swan BK, Landry ZC, Woyke T, DeLong EF, Stepanauskas R, Giovannoni SJ.** 2014. Single-cell enabled comparative genomics of a deep ocean SAR11 bathytype. ISME J.

34.  **Altschul SF, Madden TL, Schäffer AA, Zhang J, Zhang Z, Miller W, Lipman DJ.** 1997. Gapped BLAST and PSI-BLAST: a new generation of protein database search programs. Nucleic Acids Res 25:3389–3402.

35. **Camacho C, Coulouris G, Avagyan V, Ma N, Papadopoulos J, Bealer K, Madden TL.** 2009. BLAST+: architecture and applications. BMC Bioinformatics 10:421.

36. **Dongen S van.** 2000. Graph clustering by flow simulation. s.n.], S.l.

37.  **Edgar RC.** 2004. MUSCLE: multiple sequence alignment with high accuracy and high throughput. Nucleic Acids Res 32:1792–1797.

38. **Edgar RC.** 2004. MUSCLE: a multiple sequence alignment method with reduced time and space complexity. BMC Bioinformatics 5:113.

39. **Darriba D, Taboada GL, Doallo R, Posada D.** 2011. ProtTest 3: fast selection of best-fit models of protein evolution. Bioinformatics 27:1164–1165.

40. **Stamatakis A.** 2014. RAxML version 8: a tool for phylogenetic analysis and post-analysis of large phylogenies. Bioinformatics 30:1312–1313.

41. **Wu M, Chatterji S, Eisen JA.** 2012. Accounting For Alignment Uncertainty in Phylogenomics. PLoS ONE 7:e30288.

42. **Castresana J. 2000.** Selection of conserved blocks from multiple alignments for their use in phylogenetic analysis. Mol Biol Evol 17:540–552.

43. **Talavera G, Castresana J. 2007.** Improvement of Phylogenies after Removing Divergent and Ambiguously Aligned Blocks from Protein Sequence Alignments. Syst Biol 56:564–577.

44.  **Moreno-Hagelsieb G, Latimer K.** 2007. Choosing BLAST options for better detection of orthologs as reciprocal best hits. Bioinformatics 24:319–324.

45. **Grote J, Thrash JC, Huggett MJ, Landry ZC, Carini P, Giovannoni SJ, Rappé MS.** 2012. Streamlining and core genome conservation among highly divergent members of the SAR11 clade. MBio 3:e00252–12.

46. **Medini D, Donati C, Tettelin H, Masignani V, Rappuoli R.** 2005. The microbial pan-genome. Curr Opin Genet Dev 15:589–594.

47. **Tettelin H, Masignani V, Cieslewicz MJ, Donati C, Medini D, Ward NL, Angiuoli SV, Crabtree J, Jones AL, Durkin AS, others.** 2005. Genome analysis of multiple pathogenic isolates of Streptococcus agalactiae: implications for the microbial “pan-genome.” Proc Natl Acad Sci U S A 102:13950–13955.

48. **Soding J.** 2005. Protein homology detection by HMM-HMM comparison. Bioinformatics 21:951–960.

49. **The UniProt Consortium.** 2014. Activities at the Universal Protein Resource (UniProt). Nucleic Acids Res 42:D191–D198.

50. **Tatusova T, Ciufo S, Fedorov B, O’Neill K, Tolstoy I.** 2014. RefSeq microbial genomes database: new representation and annotation strategy. Nucleic Acids Res 42:D553–D559.

51. **Edgar RC.** 2010. Search and clustering orders of magnitude faster than BLAST. Bioinformatics 26:2460–2461.

52. **Price MN, Dehal PS, Arkin AP.** 2010. FastTree 2–approximately maximum-likelihood trees for large alignments. PloS One 5:e9490.

53.  **Michael Droettboom, John Hunter, Eric Firing, Thomas A Caswell, Darren Dale, Phil Elson, Jae-Joon Lee, Damon McDougall, Andrew Straw, Benjamin Root, Jouni K. Seppänen, Ryan May, Varoquaux, Tony S Yu, Charlie Moad, Jens Hedegaard Nielsen, Christoph Gohlke, Peter Würtz, Paul Ivanov, Jeff Whitaker, Matt Giuca, Paul Hobson, mmetz-bn, James Evans, Cimarron, Ian Thomas, dhyams, Thomas Hisch, NNemec, jaytmiller.** 2014. matplotlib: v1.4.2.

Figure S1. 16S ribosomal RNA-based phylogenetic tree showing the position of the 5 SAR202 SAGs within the phylum *Chloroflexi.* The tree shows 5 distinct groups within the SAR202 clade, the first four of which correspond to the taxonomy put forth in Morris et. al, 2004. The majority of marine SAR202 are represented by groups II and III, with the namesake of the clade being placed in group III. Group V represents a previously unreported group of SAR202 whose environmental relevance is yet to be determined. The SAR202 single-amplified genomes (SAGs) are marked in red, with 4 of the genomes belonging to the Group III SAR202 and a fifth genome belonging to Group V scale. The tree is rooted on *Streptomyces bikinensis*. The bar represents 0.2 nucleotide substitutions/per site.

Figure S2. Mean, mode, standard deviation, and power-law regression, of the distribution of conserved gene set size for all possible combinations of a given number of the Chloroflexi genomes included in our analysis. The mode closely follows the values predicted by the power-law regression, which approaches an asymptote at 71 conserved genes in the tail region.

Figure S3. Box-plot distributions of genome recovery as a function of recovered Chloroflexi-conserved single-copy marker genes, based on a rolling starting point within the concatenated genome sequence for all Chloroflexi included in our analysis. Boxes represent first and third quartiles of genome fractions recovered for a given number of recovered conserved single-copy genes. Whiskers represent IQR 1.5 (interquartile range * 1.5) of genome fractions recovered for a given number of recovered conserved single-copy genes. Red bars represent the median, and ‘+’ symbols represent outlier data points outside of the IQR 1.5. Notice the increase in observed outliers, as well as the increase in skew for distributions in the tail ends of range of recovered conserved single-copy genes.

Figure S4. Reciprocal best-blast recruitment of SAR202 contigs to the MOCA North Atlantic depth profile. Depths of recruited fragments are color-coded according to the key in the upper right.

Figure S5. Reciprocal best-blast recruitment of SAR202 contigs to the HOTS/ALOHA depth profile. Depths of recruited fragments are color-coded according to the key in the upper right.

Figure S6. The fraction of protein-coding genes in the SAR202 genomes with discernible paralogs at a number of similarity thresholds, as compared with *Ktedonobacter racemifer* and a number of other representative marine bacteria. The SAR202 genomes and *Ktedonobacter* show consistently higher levels of paralogs compared to the other organisms.

Table S1. Assembly statistics for 5 SAR202 single-amplified genomes (SAGs). Crossover point (Cp) represent the number of hours into the MDA reaction where the product reached half of its maximum dsDNA fluorescence. Only contigs larger than 2000 bp were used in our analyses.

Table S2. Reciprocal best blast recruitment of SAR202 single amplified genomes against reads from two metagenomic depth profiles. Note the highest levels of recruitment are from the deepest samples, indicating that these cells likely interact with chemical species found in most remote depths of the ocean.

Table S3. Candidate protein sequences found within the SAR202 genomes that potentially code for enzymes or enzyme subunits required by the 3-hydroxypropionate cycle.
